# Individual and family self-management of genitourinary health in hospitalized men: is there room for intervention by nursing professionals?

**DOI:** 10.1590/1980-220X-REEUSP-2025-0135en

**Published:** 2025-12-15

**Authors:** Carolina Andrade Costa, Osvaldo Conceição Portugal, Stefane Santos de Jesus Pitanga, Ariana Luiza Rabelo, Rafael Lima Rodrigues de Carvalho, Anderson Reis de Sousa

**Affiliations:** 1 Universidade Federal da Bahia, Escola de Enfermagem, Salvador, BA, Brazil.

**Keywords:** Self-Management, Urogenital System, Male Urogenital Diseases, Men’s Health, Nursing Care, Nursing Theories

## Abstract

**Objective:**

To analyze individual/family self-management of genitourinary health in hospitalized men and the nursing interventions observed.

**Method:**

Qualitative study of technological development using the Praxic Model for Technology Development. Thirty-five participants took part: 19 male patients, eight family members, and eight nursing professionals. Participant observation, the method of Reflective Thematic Content Analysis, and the Individual and Family Self-Management Theory framework were used.

**Results:**

Individual self-management is marked by the experience of hospitalization, weakened in everyday life, restrictive in terms of private hygiene care, seeking medical-centered health care of medium/high complexity, self-medicated herbal medicine, tolerance of symptoms, with worsening of the urological clinical condition. There is female family support, with results that promote well-being and nursing interventions focused on performing techniques and procedures.

**Conclusion:**

Self-management of genitourinary health by men is enhanced by the experience of hospitalization. Men resort to and rely on family support during hospitalization, especially from their partners, whose nursing interventions are not specific, individualized, or conducive to individual and family self-management.

## INTRODUCTION

Genitourinary health has been a cause for concern for global health, posing growing challenges for public health policies and health systems. The prevalence of diseases affecting the genitourinary tract has increased significantly, negatively impacting important dimensions of quality of life and well-being, with physical, emotional, and social consequences^(1,2)^.

It should be remembered that the genitourinary system comprises a complex network of anatomical and physiological components, integrating the urinary and reproductive systems, intertwining with sexual health and the expression of sexuality. Essential for the maintenance of life, this system performs key functions such as metabolism management, toxin excretion, body volume regulation, hormonal homeostasis, and sexual and reproductive functions.

Often exposed to various conditions, the genitourinary tract requires attention and evaluation in health services, which, particularly in cisgender men, there is a wide variety of already documented diseases and problems, such as prostatic hyperplasia, prostate cancer, prostatitis, genital infections, Peyronie’s disease, penile and testicular cancer, renal failure, urinary tract infections (UTI), lithiasis, urinary incontinence, sexual dysfunction, premature ejaculation, and other associated reproductive problems^(2,3,4,5)^.

Epidemiological information on genitourinary conditions is extensive, given its broad context, however, it is important to look at some data. International literature indicates that 90% of men between the ages of 50 and 80 live with one or more symptoms of lower urinary tract problems^(2)^. In Poland, of 214, 063 hospitalizations in the urology sector, 72% were male^(5)^. In Asia, urinary incontinence (UI) is identified as a risk factor for anxiety, depression, and difficulties at work^(6)^. In Brazil, a study of 375 male participants found that 51.1% had some degree of erectile dysfunction and 35.2% had some degree of lower urinary tract symptoms, significantly correlated with age and quality of life^(7)^.

Even in this scenario, a portion of the male population, often influenced by traditional standards of masculinity, is reluctant to access health services, which implies the need for strategies to expand access and management of health problems in this population^(6)^. With regard to nursing practice, the implications of genitourinary health are profound and multifaceted, with a wide range of possibilities for action, which can include everything from care provided by clinical nurses to care provided by specialized nurses. It should be emphasized that nurses play a key role in the prevention, assessment, management, and rehabilitation of genitourinary disorders. Evidence-based actions have the potential to significantly improve patient outcomes, which can be enhanced by the use of technological innovations, with a view to providing quality care^(2)^.

Taking charge of one’s own care, together with family members, is essential for the development of self-management in favor of quality health. One way to help individuals take responsibility as the main authors of their self-care is through care based on the theory of self-management. This theory, developed by nurses Polly Ryan and Kathleen Sawin, states that individuals and families who engage in self-management behaviors improve health outcomes, as there is a need to manage chronic conditions and actively engage in a lifestyle that promotes health^(8)^.

There are gaps in the literature on how men manage genitourinary health in a way that is not restricted to disease in the hospital setting. A study addressing this topic can help to raise indicators of male health behaviors as a phenomenon sensitive to nursing practice. Furthermore, it is necessary to broaden the understanding of the individual resources available and used by men when faced with hospitalization and the experience of urological disease, such as those of their family members and the involvement of nursing professionals, in order to understand the interventions performed.

Against this backdrop, the objective of this article is to analyze the individual and family self-management of the genitourinary health of hospitalized men from the perspective of the involvement of nursing professionals.

## METHOD

### Type of Study

This is a qualitative study^(9,10)^, derived from a Master’s thesis in Nursing and Health, with the aim of producing technological innovation - development of a new product, through the Praxic Model for Technology Development. To this end, it involves the completion of the Pragmatic Phase, which consists of: entry into the practical field, observation/reflection, understanding/interpretation of the lived reality, knowledge of social actors, knowledge and practices for planning solutions, through the sequence of actions: deduction; analysis; induction; and synthesis derived from field research data^(11)^. This study was guided by the research question: How do hospitalized men manage their own and their families’ genitourinary health? The study design followed COREQ guidelines.

### Location

The research was conducted in a urology ward of a federal public university hospital in Bahia, Brazil. The reference unit has 23 beds, allocated in 12 rooms, with care for patients of both sexes and gender identities—a reference for transsexual surgery, with genitourinary and gynecological demands. The ward provides nursing, medical, nutritional, physical therapy, bioimaging, and laboratory services. The main diseases and conditions found were: prostate hyperplasia, malignant neoplasms of the bladder, penis, prostate, and renal lithiasis.

### Population and Selection Criteria

The study participants were adult/elderly men hospitalized with genitourinary needs, family members/companions, and nursing professionals. The selection criteria were as follows:

- Men over 18 years of age with genitourinary health needs, treated at the hospital’s urology unit. Exclusion criteria: Men newly admitted to the study unit, presenting hemodynamic instability that makes it impossible to conduct the interview;
- Family members/companions - being a family member and/or caregiver during hospitalization. Exclusion criteria: being a visitor;
- Nursing professionals - working in the urology ward under investigation. Exclusion criteria: being on vacation or on leave from occupational duties.

There was an express refusal by one patient due to pain, two companions because they did not feel comfortable conducting the interview, and three professionals due to lack of time.

### Data Collection

Data collection began in the period from July to September 2024. The research team consisted of undergraduate, master’s, and doctoral students and doctoral researchers with experience in the subject/methodology, who had undergone training in data collection and had no direct connection with the participants. Through participant observation^(12)^, guided by a specific instrument containing the following topics: 1. Characteristics of the service’s operation; 2. Profile and demands of the clientele (attitudes, behaviors, and practices of self-management of genitourinary health); 3. Professional performance/practice of the nursing team; 4. Interprofessionality, validated internally by the research group and piloted on the first day in the field for three months. The aim was to establish relationships with the social actors observed, to gain an in-depth understanding of the phenomenon and nursing interventions. Twenty-one field observation scripts were completed.

In the second stage, individual interviews were conducted in single meetings, with an average duration of 50 minutes, totaling 31 hours of audio material with patients/family members and nursing professionals. For this purpose, a semi-structured instrument was used, validated internally by members of the research group and piloted with a group of five participants. The instrument consisted of closed questions related to sociodemographic, occupational, and health characteristics (adult men hospitalized), as well as open questions related to the object of investigation, namely: Men - Tell us how you manage your own genital and urinary health care; Family members - Describe how you participate in managing the health care of the man you are accompanying; Professionals - Tell me how you intervene in the management of men’s genitourinary health and the relationship with their family members.

The interviews were concluded when the methodological criteria of Reflective Thematic Analysis were met, namely: recognition of the meanings generated from the interpretation of the collected data^(13)^. The recorded material collected was transcribed in its entirety, manually coded, organized, systematized, and identified in a single file with the help of Google Drive^®^ and an Excel^®^ spreadsheet to constitute the analysis corpus. The integrity, duplication, and incompleteness of the data were verified.

### Data Analysis and Processing

The Reflective Thematic Content Analysis method was adopted, performed in six phases: (1) Familiarization with the data - immersion through reading and rereading the collected data to identify initial ideas and extract labels (157); (2) Generation of initial codes - systematic production of initial codes (58); (3) Search for themes - codes were grouped into possible themes (56); (4) Review of themes, refinement of identified themes, verifying representativeness and internal consistency; (5) Definition and naming of themes, refinement of themes, assigning clear names and descriptions; (6) Production of the report, preparation of the scientific article^(14,15)^. Steps were taken to achieve reliability in qualitative research: internal validation by the research team and members of the research group, external assessment by experts in the field and by the participants^(16)^.

The Theory of Individual and Family Self-Management was used to interpret the findings, which explains self-management as a multidimensional and complex phenomenon that affects individuals and families. Self-management has been used to refer to three phenomena: the process, related to self-regulation skills to manage chronic conditions or risk factors, such as goal setting, self-monitoring, and reflective thinking. The second phenomenon: program/interventions, which relates to empowering people to take responsibility for managing their own diseases as well as engaging in health promotion, prevention, and recovery activities. The third and final phenomenon is the results achieved after the individual’s involvement in self-management actions, related to the process of improving or stabilizing their health condition^(8)^.

### Ethical Aspects

The project was cleared under CAAE Opinion: 76017423.3.3001.0049 and No.: 6.821.769/2024, in accordance with Resolutions 466/2012, 510/2016, and 580/2018 of the National Health Council. A Free and Informed Consent Form was applied for the consent of each group of participants. To ensure the anonymity of the participants, identification was used by means of acronyms: H for male, F for family member, and PE for nursing staff, followed by the category exercised, followed by the interview order number: M01, F05, and PE - Nurse.

## RESULTS

### Characterization of Participants

The study consisted of 19 hospitalized men, eight family members/companions, and eight nursing professionals, totaling 35 study participants.

Hospitalized adult and elderly men (19): average age of 59, mostly self-declared black (11), marital status married (12), cisgender gender identity (19), heterosexual sexual orientation (19), level of education - complete high school (6), with only one participant having completed higher education. Regarding their situation in the labor market, most were retired (14), with two of them being farmers. Regarding average salary income, they reported “having income and contributing to the family’s livelihood” (14). They live in their own homes (18), all with access to electricity and a bathroom, however, 15 reported having running water and 13 access to basic sanitation, living in urban areas (13) and rural areas (6), in the presence of their emotional-marital partners (16). Most had access to the Internet (14) and a cell phone (16).

Family members/companions (8): women, including 6 wives, 2 daughters, black (5), heterosexual (8), cisgender (8), unemployed (3), followed by retired (2). Nursing professionals (8): female (7), black (5), cisgender (8), heterosexual (8), higher education (6) - Nurse and technical training (2), postgraduate education (5), however, none in the field of Nursing in Urology/Nephrology. Five of these had only one job, with a professional career of more than 10 years of training (7), no previous professional experience in the field of urology, working a 36-hour week.

### Empirical Findings of the Investigated Phenomenon

The empirical results were presented within the theoretical framework, considering the following aspects: Constituent elements of Self-Management Theory; Substrate of empirical data coding and evidence of empirical findings, regarding: self-management context; self-management processes; proximal and distal outcomes; and apparent interventions.

### Individual Dimension of Self-Management

#### Initial Generating Theme: The Context of Self-Management of Genitourinary Health

The context of self-management of genitourinary health performed by adult men in hospitalization is linked to basic private hygiene care, justified by the absence of genital diseases, family history of diseases and genitourinary diseases, especially among other men in the family, the maintenance of a link with an environment considered healthy, the evolution of worsening clinical conditions, leading them to interventions, procedures, and hospitalization, and the perception of support from family members.

Family members were involved in a context in which the self-management of genitourinary health of hospitalized men requires them to migrate from their city, travel to therapeutic appointments in the urban center, at a referral hospital, having experienced access to other health services available in the care network as companions, being significantly composed of women, whether they are romantic partners, daughters, or other women in the family circle. These women seek to cooperate in promoting a healthy family environment, including the hospital nurse, supporting the urological treatment measures employed by the health team, as well as caring for nutrition and the use of prescribed medications. In addition, they expressed their perception of the significant person in relation to health and the difficulties they experience in performing the role of companion. Thus, Chart 1 presents the theoretical framework of the context for individual and family self-management.

**Chart 1:** Theoretical framework of the context of individual and family self-management of the genitourinary health of hospitalized men – Salvador, BA, Brazil, 2025.

Constituent elements of the Theory of Self-Management	Substrate for the encoding of empirical data	Empirical findings
**Context:** Protective factors: understanding of private hygiene; correlation between the use of soap for private hygiene; rural, domestic, and work environments; presence and readiness of family members; Risks: lack of progress in private hygiene care due to the absence of disease/illness; presence and recurrence of urinary tract infections; history of genitourinary disease in the family - prostate cancer		
**Condition – specific factors; individual/family perception** (Complexity of condition and treatment, trajectory, stability of condition, and transitions)	Theme: Intimate hygiene using regular soap; Theme: Basic private hygiene care in the absence of genital problems; Theme: Washing and use of products for penis hygiene; Theme: History of genitourinary events throughout life; Theme: Progression of genitourinary health problems; Theme: Submission to procedures to solve urinary problems; Theme: Multiplicity of genitourinary diseases and conditions; Theme: Experience of critical genitourinary health events; Theme: Experience of urinary/micturition complications; Theme: Trajectory involving interstate migration; Theme: Family accompaniment during transfer to the hospital; Theme: Family accompaniment in specialized outpatient service	[...] *I don’t use any specific hygiene products. I take normal precautions, such as using soap, especially since I don’t have any genital problems. I’ve never had any diseases before.* (H01); [...] *my health problem has been going on for many years. I had surgery many years ago, and I still have many episodes today. I have had many urinary tract infections and went to the machine to “break the stones”* (refers to the lithotripsy procedure). (H05); [...] *there are days when I go to the bathroom four to five times to urinate. The problem is that urine drips when I pee.* (H07); [...] *what affected me was having to go to the bathroom several times to urinate.* (M08); [...] *we were referred by the municipal service where we live and then we came here* (referring to the state referral hospital)*.* (F05); [...] *he had access to the polyclinic in my region where I live.* (F05)
**Physical and social environment** (Access to healthcare, transportation, culture, social capital)	Theme: Expanding access to urological health services; Theme: Addressing difficulties in hospital care in urology; Theme: Maintaining coexistence in rural environments; Theme: Maintaining work activities; Theme: Promotion of a healthy environment by family members; Theme: Family support in urological treatment.	[...] *the hospital needs to open its doors so that we men don’t have difficulties, preventing them from giving up on taking care of themselves because they face difficulties in getting an appointment, scheduling the procedure, finding a place in the hospital, and transportation to get there.* (H01); [...] *I plan to continue living in the countryside, doing my work,* eating well *to live longer.* (H03); [...] I take care of the food, the house, I always try to keep the environment clean, healthy, hygienic, and the clothes clean. (F02); [...] when the health problem arose, we started to seek treatment in the nearest city, but we couldn’t resolve it, we had to travel and be referred to the hospital in the capital. (F05)
**Individual and family factors** (Stages of development, learning ability, literacy, family structure and functioning, self-management skills)	Theme: Understanding the impact of sexually transmitted infections on genitourinary health; Theme: Perception that men have multiple sexual partners; Theme: Limited knowledge of genitourinary diseases and conditions; Theme: Healthy eating as a strategy for managing genitourinary function; Theme: Use of genitourinary hygiene measures and perception of achieving good results; Theme: Attribution of causal relationships between urinary complications and kidney stones; Theme: History of kidney and genitourinary disease among male family members; Theme: Paternal mortality and its relationship to prostate cancer; Theme: Family support and the role of romantic partners; Theme: Family support in providing food and medical care; Theme: Family perception of the health condition of the significant person; Theme: Experiencing difficulties in acquiring knowledge to care for family members.	[...] *I know that some communicable diseases can cause problems in the genital and urinary organs, such as cancer, in the case of men who have many sexual partners.* (H01); [...] *I have heard about urinary tract infections* [...] *my father had a urinary tract infection.* (H03); [...] *I try not to eat fatty foods, not to gain weight. In addition, I take care with the urinary catheter, avoiding touching it on the floor, leaving it hanging to prevent bacteria from reaching the bladder.* (H04); [...] I wash my penis thoroughly and clean it after urinating [...] people say that kidney stones are caused by not drinking enough fluids, and I drink plenty of water [...] I have two brothers who use kidney drains. (H05); [...] *I need to take care of myself, try to be clean* [...] *before he died, my father had prostate disease, his prostate was altered to the point that he couldn’t urinate.* (H06); [...] *I have the support of my family. My wife offered and was willing to stay with me and take care of me. We have a good relationship; despite the short time we have been together.* (H07); [...] *I think I do my private hygiene correctly.* (H08); [...] *I drink little water, but I will drink more. I use soap for private hygiene* [...] *two brothers had prostate* problems. (H09); [...] *I have a large family and for this reason I always have someone to count on to support me in taking care of my health, especially now that I am hospitalized and needed surgery.* (H10); [...] *I give him his medication, I prepare his meals, but he can bathe and take care of other things on his own.* (F03); [...] *I was not aware of his health problem.* I only *found out after he was hospitalized.* (F06); [...] *he has been diagnosed with kidney and prostate disease, but the prostate does not require intervention at this time. I have heard of kidney disease, but I confess that I do not have enough knowledge to care for him.* (F07)

**Figure 1:**
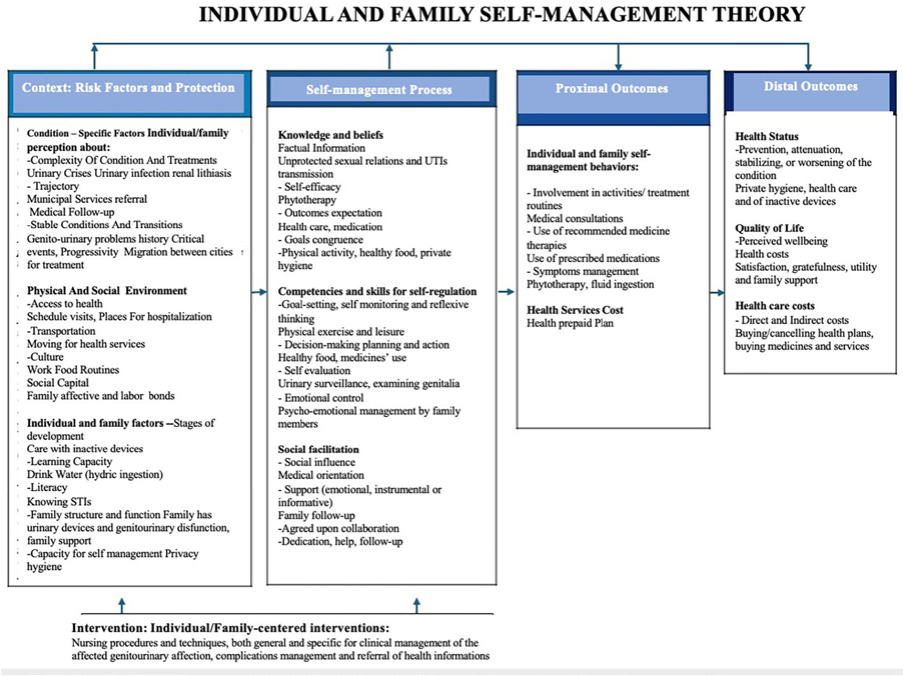
Explanatory model of the application of the Individual and Family Self-Management Theory of genitourinary health in hospitalized adult men. Salvador, BA, Brazil.

Source: Adapted from Ryan, Sawin, 2009.

The process of self-management of genitourinary health employed by adult men hospitalized expressed (Figure 1), knowledge and beliefs related to the causes of genitourinary diseases, with an emphasis on sexually transmitted infections (STIs), the use of phytotherapy, often recommended by close friends, used on their own and without the recommendation of a health professional.

Goals were established for the current hospitalization and the future, with a view to improving physical activity, diet, medication use, and self-care. In addition, they expressed mechanisms of self-control of genitourinary symptoms and complaints, such as self-inspection and monitoring of urinary elimination. They also brought up reflective thoughts about their current health condition and the possibilities of reframing habits and practices of genitourinary health care, improving and/or leveling their health status. To support self-management, family members referred to God, expressing perceptions about the relationship between the family member and their health care, permeated by the context of worsening genitourinary health and the emergence of complications, linked to the support strategies that are granted to men. Furthermore, family members had to deal with the apprehension of surgery and the search for ways to facilitate self-management, as they expressed affection for the sick family member, demonstrating dedication and attributing value to caring for the other, most of whom were affective-significant partners. Thus, Chart 2 presents the theoretical framework of the self-management process for individual and family self-management.

**Chart 2:** Theoretical framework of the process of individual and family self-management of genitourinary health in hospitalized men – Salvador, BA, Brazil, 2025.

Constituent elements of the Theory of Self-Management	Substrate for the encoding of empirical data	Empirical findings
**Self-management process**		
**Knowledge and beliefs** (Factual information, self-efficacy, outcome expectancy, goal congruence)	Theme: Attributing masculinity to genitourinary health; Theme: Adhering to herbal remedies recommended by close friends and family; Theme: Adopting avoidance behavior to prevent genitourinary health complications; Theme: Concept of health beyond illness and the expanded search for medical professionals from different specialties; Theme: Assigning age groups as a benchmark for establishing self-management measures for health and disease; Theme: Seeking out a urologist; Theme: Referring to God as beneficial and a source of comfort, demonstrating service to Him.	[...] *there are many men who don’t use condoms during sex, they do whatever they want. That’s why problems like HTLV, HIV, syphilis, and all that arise, compromising genital and urinary function.* (H02); [...] *make stone-breaking tea, mandacaru, or whatever else people recommend to me.* (H05); [...] I’m trying to take care of my health so that problems don’t accumulate, otherwise it will be much worse, I can’t stop. (H07); [...] health is prevention, I need to take care of myself even if I’m not sick, go to the doctor, not only the urologist but also other specialists. (H08); [...] I take normal precautions. If I need to use medication, I use it; (H10); [...] from the age of 40 or 45, men have to see a urologist [...] *since I got sick, I have tried to be a servant of God. I had problems in the past that hurt me and didn’t allow me to feel well. That’s when I heard the word of God, which served as comfort.* (H11). [...] *thank God I was fine when I saw him return from surgery* [...] *I will continue to ask God to grant him health.* (F05)
**Self-regulation skills and abilities** (Goal setting, self-monitoring and reflective thinking, decision making, planning and action, self-assessment, emotional control)	Theme: Pursuing longevity through healthy eating, physical activity, and leisure; Theme: Reflecting on oneself and one’s current health status and future projections; Theme: Compliance with medical therapy related to medicalization; Theme: Assigning health self-assessment parameters; Theme: Adopting behaviors that promote water consumption; Theme: Identifying unpleasant urinary symptoms; Theme: Inspection of the genitals with mention of the penis and testicles; Theme: Explaining behavioral characteristics and personality traits related to health care; Theme: Expressing tolerance for pain and other symptoms; Theme: Revealing the perception of the family member’s relationship with health; Theme: Revealing the severity of the family member’s genitourinary health problem and the support strategies provided.	[...] *I try to exercise, read, see what’s best.* (H01); [...] *if I don’t take care of myself, who will?* (H06); [...] *I eat well, take my medication, do what the doctors tell me to do* [...] *if I don’t take care of myself now, what about tomorrow? Today I have 90% urinary flow. If I leave it to take care of tomorrow, how will I be?* (H04); [...] I’m taking care of myself, nowadays we need to take care of ourselves, the doctor ordered it. [...] *I’m alert to drink plenty of water. What I usually do now is follow the advice to drink plenty of fluids. I notice a bad smell in my urine when I don’t drink enough water.* (H05); [...] *I check how my testicles are doing.* (H07); [...] *my relatives usually live to be 100 years old, and I also intend to reach that age without needing anyone’s help.* (H10); [...] sometimes I am stubborn, I delay going to the health service [...] I am a little hard-headed, I felt pain and stayed at home for a long time resisting, I was not very disciplined, but now I need to maintain discipline [...] with everything that has happened to me, it has opened my eyes, my body gave me a sign, the body gives us signs, doesn’t it? *I stayed at home for a long time wondering: will this pain go away? Now I’m reflecting on myself, on my health, let’s say... good health is when you’re 100%. I see that mine is at 40%.* (H11); [...] *he has difficulty going to the doctor, he doesn’t like it. When the family found out he had this problem, we immediately started looking for ways to find a doctor.* (HA05).
**Social facilitation** (Social influence, support—emotional, instrumental, or informational, negotiated collaboration)	Theme: Family support during urological hospitalization; Theme: The role of wives as companions in the hospital unit; Theme: Understanding health information provided by medical professionals; Theme: Between apprehension about surgery and relief at having a family member nearby; Theme: Family dedication to caregiving; Theme: Valuing the care provided to a spouse	[...] *my family has always been there for me. My wife accompanied me here to the hospital.* (H07); [...] *about diseases such as cancer and urinary tract infections, I always see doctors talking about them.* (H11); [...] *I felt nervous at the time of his surgery, but everything was a success and today he is well, hospitalized, receiving my support.* (HA05); [...] I dedicate all my time to him, taking care of him all day, every day, with food, clothes, and other things. I remember his medications and accompany him to the doctor. (HA06); [...] he is my husband, I have to take care of him and accompany him in the best way possible, and in the best way I can. (HA07)

The results emerging from the self-management of genitourinary health employed by adult men hospitalized revealed, in a proximal dimension, the search for medical assistance, with the presence of emotional and marital support, the appearance of nurses as promoters of health education, access to urgent and emergency health services, and adherence to Integrative and Complementary Practices linked to phytotherapy. Given the context of the distal outcomes obtained in the experience of self-management of genitourinary health, family members expressed an affinity for caring and a feeling of well-being in performing this exercise, which is intertwined with the manifestation of fatigue, overload, and concerns for the hospitalized family member (Chart 3), which gives rise to nursing interventions, which were concentrated on clinical management and the execution of nursing procedures, with limited educational intervention and promotion of individual and family self-management (Chart 4).

**Chart 3:** Theoretical framework of the proximal and distal outcomes of individual and family self-management of the genitourinary health of hospitalized men – Salvador, BA, Brazil, 2025.

Constituent elements of the Theory of Self-Management	Substrate for the encoding of empirical data	Empirical findings
**Proximal and distal outcomes**		
Proximal outcomes – individual and family self-management behaviors (Engagement in treatment activities/regimens, use of pharmacological therapies, symptom management)	Theme: Medical monitoring as a genitourinary treatment activity/regimen; Theme: Female presence as a promoter of health for men with genitourinary diseases; Theme: Nurses as professionals who teach, guide, and provide training; Theme: Self-medication and use of herbal medicines as practices; Theme: Seeking medical professionals as a secondary option after attempts at self-management at home; Theme: Seeking urgent and emergency services; Theme: Medical professionals as the first point of contact; Theme: Use of medication as a therapeutic resource for symptom management	[...] *in my case, it was medical follow-up for me and my wife. I seek medical assistance.* (H01); [...] *I have always sought doctors to guide me. It is always good to have guidance from doctors* [...] *the nurse teaches me and guides me so that I can do the training here at the hospital.* (H02); [...] I took medicine on my own, used a plant and made my own tea. Not medicine. I took a boldo leaf, cooked it, and drank it for relief. When the situation got bad, I sought medical attention. (H03); [...] I seek care because the doctor told me to. (H04); [...] I drink liquids, tea made from quebra pedra leaves and macaco do brejo cane [...] when that doesn’t work, I go to the doctor. (H05); [...] I tried to find a doctor, and that happened at the UPA^*^. *I take medicine such as dipyrone and boldo tea, holy grass, lemon grass, when I am at home.* (H06); [...] *I went straight to the doctor at the clinic.* (H07); [...] *I have needed to use medication on my own.* (H10). ^*^24-hour Emergency Care Unit
Proximal outcomes – cost of healthcare services (The cost associated with the use of healthcare)	Theme: Adoption of measures considered healthy in the face of the impossibility of affording other resources; Theme: Need for pharmaceutical assistance. Theme: Disbursement of financial resources with health insurance.	[...] *Today, I look for the best possible way to live a healthy life, because I cannot afford health insurance.* (H09); [...] *I have needed to take medication, and today I pay for health insurance*. (H10).
**Distal outcomes – health status** (Prevention, mitigation, stabilization)	Theme: Establishment of preventive genital care; Theme: Adoption of hygiene care with urinary devices; Theme: Use of herbal medicines in the form of teas as a solution for symptom relief; Theme: Seeking medical assistance with regularity; Theme: Practice of self-medication as a therapeutic resource.	[...] *I am in good health because I maintain good hygiene; my penis is always clean, as is my urinary catheter.* (H04); [...] *the solution to my kidney problem was to drink tea. When I drank tea, it solved the problem and made me feel good.*(H06); [...] *I see a doctor now, but in the past, when I had pain, I would try to take medicine.* (H09).
**Distal outcomes – quality of life** (Perceived well-being, healthcare costs)	Theme: Satisfaction in seeking to improve health and obtaining help from others; Theme: Perception of well-being when taking care of one’s health; Theme: Feeling of gratitude linked to family ties; Theme: Feeling of optimism and usefulness; Theme: Feeling of fulfillment linked to family cohesion; Theme: Perception of prioritizing one’s health; Theme: Affinity for caring and feeling of well-being in managing the health-illness of a family member.	[...] *I am satisfied with seeking improvement. I am maturing and seeking what is best for me.* (H01); [...] *I feel satisfied to have people helping me.* (H03); [...] *I feel good, it does me good, and I need to take care of myself for my well-being.*(H05); [...] *the teas I drank made me feel good. I feel good taking care of my health. I am grateful for the family I have, which makes me feel good.* (H06); [...] *it’s great to take care of myself and know that I’m useful.* (H09); [...] *I feel fulfilled with the family support I have to take care of my health.* (H10); [...] *I feel good because I’m taking care of my health, which is now a priority.* (H11); [...] *when I take care of him, it’s because I like to see him feeling good, and that makes me feel good too.* (HA02); [...] *I feel good because I get along well with him, thank God, he’s a great husband, so I have to take care of him.* (A05)
Distal outcomes – healthcare costs (Direct and indirect costs)	Theme: Cancellation of measures to maintain health care; Theme: Disbursement of financial resources for health maintenance; Theme: Fatigue, overload, and concerns experienced by family member	[...] *I had health insurance, but it was expensive, so I canceled* it. (H09); [...] *I pay for health insurance so he can come visit me*. (H10); [...] *it’s exhausting, because my father needs constant care*. *We were concerned about his diagnosis and health situation. And I was particularly anxious to accompany him.* (HA03); [...] *I have to do it for him, sometimes I feel very tired, overwhelmed.* The surgery took a long time, and I was worried. (A04); [...] I was afraid, and the longer it took for him to return from the operating room, the more desperate I became. He returned during the night (A05).

#### Professional Dimension of Self-Management – Nursing Professionals

**Chart 4:** Theoretical framework for interventions focusing on individual and family self-management of genitourinary health in hospitalized men – Salvador, BA, Brazil, 2025.

Constituent elements of the Theory of Self-Management	Substrate for the encoding of empirical data	Empirical findings
**Interventions**		
**Interventions focused on the individual and family**	Theme: Performing nursing techniques and procedures; Theme: Controlling urinary elimination through bladder irrigation; Theme: Unblocking the catheter; Theme: Physical examination of the genital tract; Theme: Control of fluid balance; Theme: Control of hemorrhagic episodes; Theme: Establishing dialogue to expand health knowledge among male patients.	[...] *medications, changing equipment and devices.* (P01); [...] *dressing infected wounds, using products such as activated charcoal alginate* [...] *bladder irrigation, bleeding control, nursing records.* (P02); [...] *administering medication, caring for drains, eliminating secretions, bladder irrigation.* (P03); [...] *effectively controlling fluid balance and bladder irrigation.* (P04); [...] I try to talk to patients so that they are aware of their health condition. (P05); [...] I perform catheter unblocking, especially when the patient returns from the operating room [...] physical examination of the genitals. (P05).

Source: Adapted from Ryan, Sawin, 2009.

## DISCUSSION

The results of this study revealed a predominance of black and brown, cisgender, heterosexual, married individuals with different levels of education and mostly retired, with an average age of 59 years. Considering the profile of the men interviewed is essential for analyzing their individual and family self-management of genitourinary health, since these characteristics can influence the care process.

With regard to the context of self-management of genitourinary health, among the protective and risk factors, basic private hygiene care, family history of genitourinary diseases and conditions, the female figure as a promoter of care, with a focus on wives, maintenance of a healthy environment, and lack of progress in private hygiene linked to the absence of disease emerged as risk factors. The contextual factors explained in the Theory contribute to a broader understanding of how self-management is performed at the individual level and within the family dyad or family unit. Such contextual factors can affect self-management and compromise the process, given the contextual and procedural dimensions that exist in health management, especially when living with affected genitourinary functions. When nursing interventions consider the context, they can contribute to reducing health risks and promoting conditions favorable to self-management, which can be widely evidenced in our findings^(8)^.

In relation to the widely mentioned private hygiene, findings in the literature indicate that male knowledge associated with body care is often associated with the notion of body hygiene, with relevant importance to the genital organs, associated with continuous inspection of these^(17)^. However, it is important to highlight that genitourinary care should not be limited to the genitals alone, but should be broader and integrated with general health.

In this study, it was evident that men did not appear as caregivers, while women appeared as promoters of men’s health and protagonists of family self-management, which can be correlated with sociocultural issues, in which caregiving has little social recognition and is strongly feminized^(18)^. Women, often wives and daughters, are jointly responsible for managing care, especially in the presence of a sick loved one, and are permeated by feelings that involve well-being from caring for and managing the health and illness of their family member, but also by moral responsibility, obligation, and difficulties with overload, fatigue, and concerns for their hospitalized family member^(18)^.

In the process of self-management of genitourinary health, the influence of knowledge and beliefs was evident, with constant use of phytotherapy, self-medication, and the establishment of goals with a view to improvement. The findings of this study present the well-known components of the self-management process, which can support the understanding of the results, whether they are proximal or distal to genitourinary health management^(8)^. The use of herbal medicines, often recommended by neighbors, friends, and family members, presents complex nuances. The use of medicinal plants to promote health and/or complement treatments for certain conditions should be viewed with caution, as their use is not without risks^(19)^. These findings can be explained by the Theory of Self-Management, whereby nursing interventions aimed at the self-management process contribute to improving health knowledge and beliefs, as well as helping to increase the adoption of self-regulation behaviors by individuals, facilitating socialization^(8)^.

Furthermore, although Brazil has regulations through the National Policy on Medicinal Plants and Phytotherapies and includes it in the Integrative and Complementary Practices of the SUS, studies indicate misguided knowledge in several important aspects, such as the adverse effects of this therapy, lack of knowledge about chemically active substances, and lack of knowledge about drug interactions^(19,20,21)^. In this scenario, it is essential to explore multi- and interdisciplinary knowledge involving popular and scientific knowledge in the search for strategies that promote well-being and reduce or eliminate the possibility of harm to the user and worsening of a particular condition^(19)^.

In the proximal and distal outcomes, the medical figure was used as a reference, the secondary search for a medical professional, the expenditure of financial resources, and difficulty in accessing specialized services. It should be noted that in the Theory of Individual and Family Self-Management, social influence is conceived as a message or dialogue in which people in positions of authority, who are respected and have specialized knowledge, advise and encourage healthy behaviors^(8)^. Thus, the repeatedly mentioned physician fits into this role, since he or she is seen as the professional of first choice and as a driver of care, in which some patients perform certain health practices solely because they were advised by the physician, which may be a reflection of physician-centered health care. It also reflects the importance of the doctor-patient relationship, given its critical role in the prevention, diagnosis, and treatment of disease, as well as in therapeutic success^(19,22)^.

With regard to the search for health services, there is a close relationship with traditional patterns of masculinity. Sociocultural norms play a significant role in men’s reluctance to seek health services, often due to fear of appearing weak, stigma, and discrimination, in addition to the scarcity of male professionals^(23,24)^. Also noteworthy is the presence of ageism in the discourse, which cites age limits as a benchmark for establishing self-management measures for health and illness. It should be noted that this phenomenon can be influenced by age, gender, education, and fear of death^(25)^.

Access to health services is crucial for therapeutic outcomes. This study highlighted obstacles that impact this access, such as long therapeutic journeys undertaken by men and their families who live in municipalities far from the capital, barriers to accessing services due to the complexity of scheduling appointments and procedures, and the availability of hospital beds. Corroborating these findings, a study that analyzed barriers to access in five regions of Brazil highlighted difficulties related to geographical accessibility associated with the unavailability of specialized consultations in the Health Region itself, insufficient hospital beds, prolonged waiting times, and poor or non-existent integration of services^(26)^. Added to this is the fact that certain genitourinary problems, such as incontinence, can have multidimensional impacts, affecting not only the quality of life of patients, but also the structure, process, and results of care^(27)^.

The findings reveal that the professional dimension of self-management with regard to the work of nursing professionals was focused on the execution of care techniques and procedures, without the appearance of nursing interventions focused on education and the application of technological resources, as in the case of the indication of applications available for the management of genitourinary health problems resulting from prostate cancer and radical prostatectomy surgeries^(28)^. Self-management theory can be useful in guiding individual and family-centered nursing interventions, as they have a positive impact on self-management of health, especially when they consider the context and process^(8)^. The same applies to diagnostic/therapeutic support resources for incontinence provided by nurses^(29)^. Therefore, the establishment of measures and programs focused on autonomy, skills, goal setting, care transition, rehabilitation, preparation, and planning for hospital discharge, as well as the scarcity of specific interventions for the promotion and maintenance of genitourinary health in the hospital context surveyed and with implications for self-care^(30)^.

It should also be noted that the nursing professionals surveyed had no training or specialization in the area of Urology Nursing, which may imply a limited scope of practice and weakening of nursing care in meeting the expanded needs of genitourinary health. It is urgent and necessary to make efforts in the field of training in Men’s Health Nursing, Nephrology Nursing, and Urology, as well as to strengthen nursing work processes, in the application of the Nursing Process and Nursing Theories that support the clinical decision-making of nursing teams working in this segment. However, caution is advised in understanding the essential concepts and constructs of the dimensions of self-management of genitourinary health by men, considering that the results achieved by men can be easily affected by the dimensions of context and process, which will require nursing professionals to invest more in clinical nursing assessment (medical history, clinical examination) – reduction of morbidity and mortality, control of metabolic rates, prevention and management of chronic conditions, accurate use of medications, with a view to improving health status through self-management^(8)^.

Likewise, nursing professionals working in other areas of the Primary Health Care network, as corroborated in a previous study that highlights the contributions of nurses in the treatment of Lower Urinary Tract Dysfunction^(29)^. In this sense, it is recommended to pay attention to what the Self-Management Theory points out when it indicates that constructions based on context, process, and results combined in a unique way can imply substantial advances for the clinical practice of self-management of chronic disease by affected men, making them more engaged in adopting specific behaviors in relation to health and genitourinary disease^(8)^.

This study presents potentialities and implications for the production of knowledge and its translation and for professional practice in Nursing: it analyzes and explains the phenomenon of self-management; it presents particular and unique aspects of gender/masculinities in the relationship between care and self-management of health by men; it strengthens clinical-social studies in the context of hospital care; it explores genitourinary health needs as a focus and research agenda; it lists the constituent elements of self-management, which are useful for directing/guiding the clinical/diagnostic reasoning of nursing professionals and directing the nursing team in the provision of genitourinary health care. In addition, the practical potential of using a Medium-Range Nursing Theory, which proved to be appropriate for the specificity investigated, can be better explored in future investigations and in care and educational interventions^(8)^.

This study has the following limitations: the technique and method used involved different groups, which may require the use of diverse and specific resources to gain a more in-depth, explanatory, and reflective understanding of the phenomenon investigated, which may limit the capacity for abstraction, as well as the understanding of the patterns, properties, and dimensions of the data found. The fact that the research was conducted in a clinical-care hospital setting may have reduced the capacity for abstraction on the part of nursing professionals, given the distractions, multiple demands, and requirements of the work process, as well as the censorship of certain sensitive and relevant information to be collected from male participants and their family members/companions, which should be taken into consideration.

## CONCLUSION

The constituent elements of individual and family self-management were found in the experience of men with genitourinary health needs affected by hospitalization and favor advances in clinical nursing practice by revealing the context and process in which and how self-management occurs, the results that are achieved in the interaction with the family and the scope, coverage, and directions of the nursing interventions that are implemented, which explicitly indicates expansion, strengthening, and specificity. In the individual dimension, the context is marked by the experience of hospitalization, with self-management of genitourinary health weakened in everyday life. Care practices are linked to private hygiene, doctor-centered health care, and the increase in self-medicated herbal medicine alternatives. There is a search for medium and high complexity health services in the face of worsening urological clinical conditions, with the female presence as a point of reference for family support.

The self-management process is based on beliefs related to genitourinary health and disease, the establishment of goals to restore health and genitourinary functioning, permeated by self-reflection and self-assessment of health, which are added to the psycho-emotional experiences of family members who accompany them during hospitalization. The proximal and distal outcomes indicate the adoption of self-management behaviors, however, mobilized by treatment and medical-centered therapies, which have repercussions in terms of costs for themselves and for the health system, but which express a sense of improvement in quality of life on the part of the men surveyed.

Family involvement is significant in the context of self-management with regard to specific factors and perceptions about the hospitalized family member, whose family functioning expresses cohesion and support, but is permeated by difficulties in understanding the genitourinary health-disease process. There is a family belief that God grants comfort, whose self-regulation skills and abilities revealed the men’s resistance to autonomous management of their health and the worsening of genitourinary health problems, which requires the use of support strategies for men with affected genitourinary needs. The result of self-management supported by family members implies benefits for themselves, but does not point to proximal contributions to men.

## DATA AVAILABILITY

The entire dataset supporting the results of this study was published in the article itself.
