# Supplementary material for: Individual and family self-management of genitourinary health in hospitalized men: is there room for intervention by nursing professionals?
Source: Rev Esc Enferm USP. 2025 Dec 15;59:e20250135. doi: 10.1590/1980-220X-REEUSP-2025-0135en (PMC12704599; doi:10.1590/1980-220X-REEUSP-2025-0135en)
Supplement: Translation

# Translation: Automanejo individual e familiar da saúde geniturinária de homens hospitalizados: há intervenção dos profissionais da Enfermagem?

**DOI:** 10.1590/1980-220X-reeusp-2025-0135pt

**Authors:** Carolina Andrade Costa, Osvaldo Conceição Portugal, Stefane Santos de Jesus Pitanga, Ariana Luiza Rabelo, Rafael Lima Rodrigues de Carvalho, Anderson Reis de Sousa

**Affiliations:** 1 Universidade Federal da Bahia, Escola de Enfermagem, Salvador, BA, Brasil.

**Keywords:** Autogestão, Sistema Urogenital, Doenças Urogenitais Masculinas, Saúde do Homem, Cuidados de Enfermagem, Teorias de Enfermagem

## Abstract

**Objetivo:**

Analisar o automanejo individual/familiar da saúde geniturinária de homens hospitalizados e as intervenções de enfermagem evidenciadas.

**Método:**

Estudo qualitativo, de desenvolvimento tecnológico, pelo Modelo Práxico para Desenvolvimento de Tecnologias. Participaram 35 integrantes: 19 homens pacientes, oito familiares e oito profissionais da Enfermagem. Empregou-se a observação participante, o método da Análise Temática de Conteúdo Reflexiva e referencial da Teoria do Automanejo Individual e Familiar.

**Resultados:**

O automanejo individual é marcado pela experiência da hospitalização, fragilizado na vida cotidiana, restritivo à cuidados de higiene íntima, busca por assistência à saúde médico-centrada na média/alta complexidade, fitoterapia automedicalizada, tolerância aos sintomas, com piora da condição clínica urológica. Há apoio familiar feminino, com resultados promotores de bem-estar e intervenções de enfermagem centradas na realização de técnicas e procedimentos.

**Conclusão:**

O automanejo da saúde geniturinária por homens é potencializado com a experiência da hospitalização. Os homens recorrem e contam com o apoio familiar na internação, especialmente, das companheiras, cuja intervenções de enfermagem não se mostram específicas, singularizadas e promotoras da autogestão individual e familiar.

## INTRODUÇÃO

A saúde geniturinária tem sido motivo de preocupação para a saúde global, representando crescentes desafios para as políticas públicas de saúde e para os sistemas de saúde. A prevalência das doenças que afetam o trato geniturinário tem aumentado significativamente, repercutindo negativamente em importantes dimensões da qualidade de vida e bem estar, com consequências físicas, emocionais e sociais^(1,2)^.

Faz-se lembrar que o sistema geniturinário compreende uma complexa rede de componentes anatômicos e fisiológicos, integrando os sistemas urinário e reprodutor, entrelaçando-se com saúde sexual e a expressão da sexualidade. Essencial para a manutenção da vida, esse sistema desempenha funções primordiais como a gestão do metabolismo, excreção de toxinas, regulação do volume corporal, homeostase hormonal e além das funções sexuais e reprodutivas.

Frequentemente exposto a condições diversas, o trato genitu-rinário exige atenção e avaliação nos serviços de saúde, particularmente em homens cisgêneros, já que há uma vasta variedade de doenças e problemas já documentados, como hiperplasia prostática, câncer de próstata, prostatite, infecções genitais, doença de Peyronie, câncer de pênis e testículos, insuficiência renal, infecções urinárias, litíase, incontinência urinária, disfunções sexuais, ejaculação precoce e outros problemas reprodutivos associados^(2,3,4,5)^.

Informações epidemiológicas acerca das condições genitu-rinárias são amplas, dado seu contexto abrangente, entretanto, é importante a aproximação com alguns dados. A literatura internacional aponta que 90% dos homens entre 50 e 80 anos vivem com um ou mais sintomas de problemas do trato urinário inferior^(2)^. Na Polônia, de 214.063 internações no setor urológico, 72% eram do sexo masculino^(5)^. Na Ásia, a Incontinência Urinária (IU) é apontada como fator de risco para ansiedade, depressão e dificuldades no trabalho^(6)^. No Brasil, um estudo com 375 participantes do sexo masculino, apontou que 51, 1% tinham algum grau de disfunção erétil e 35, 2% apresentaram algum grau de sintomas do trato urinário inferior, correlacionado significativamente com a idade e qualidade de vida^(7)^.

Mesmo diante desse cenário, uma parcela da população masculina, frequentemente influenciada por padrões de masculinidade tradicional, mostram-se resistentes em acessar os serviços de saúde, o que implica na necessidade de estratégias para ampliar o acesso e manejo dos problemas de saúde desta população^(6)^. No que concerne à prática de enfermagem, as implicações da saúde geniturinária são profundas e multiface-tadas, com amplas possibilidades de atuação, que podem incluir desde atendimentos com enfermeiro clínico a atendimentos com enfermeiros espxecializados. Sublinha-se que enfermeiros exercem um papel primordial na prevenção, avaliação, gestão e reabilitação de distúrbios geniturinários, ações baseadas em evidências têm potencial de melhorar significativamente os desfechos dos pacientes, que podem ser potencializados pelo uso de inovações tecnológicas, com vistas a proporcionar uma assistência de qualidade^(2)^.

Assumir seu próprio cuidado, em conjunto com seus familiares, é essencial para o desenvolvimento do automanejo a favor da qualidade de sua saúde. Umas das formas que pode auxiliar a responsabilização do indivíduo como autor principal do seu autocuidado é através de um cuidado baseado na teoria do auto-manejo. Esta teoria desenvolvida pelas enfermeiras Polly Ryan e Kathleen Sawin, afirmam que indivíduos e famílias que se envolvem em comportamentos de auto manejo melhoram os resultados de saúde, uma vez que existe uma necessidade de gerenciar condições crônicas e envolvimento ativo em um estilo de vida que promova a saúde^(8)^.

Há lacunas na literatura acerca de como os homens manejam a saúde geniturinária de maneira não restritiva à doença ao agravo no âmbito do contexto hospitalar. Um estudo que aborde essa temática pode auxiliar a levantar indicativos dos comportamentos de saúde masculina enquanto fenômeno sensível à prática da Enfermagem. Outrossim, é necessário ampliar a compreensão acerca dos recursos individuais existentes e empregado pelos homens quando estão diante de uma hospitalização e da experiência da enfermidade urológica, tais como de seus familiares e da inserção dos profissionais do campo da Enfermagem, na direção de apreender as intervenções desempenhadas.

Frente ao exposto o objetivo deste artigo é analisar o auto-manejo individual e familiar da saúde geniturinária de homens hospitalizados na perspectiva da inserção dos profissionais do campo da enfermagem.

## MÉTODO

### Tipo do Estudo

Estudo qualitativo^(9,10)^, derivado de dissertação de Mestrado em Enfermagem e Saúde, com finalidade de produzir inovação tecnológica - elaboração de novo produto, através do Modelo Práxico para Desenvolvimento de Tecnologias. Para tanto, trata-se do cumprimento da Fase Pragmática, configurada por: entrada em campo prático, observação/reflexão, compreensão/interpretação da realidade vivida, conhecimento de atores sociais, saberes e práticas para o planejamento de soluções, mediante a sequência das ações: dedução; análise; indução; e síntese derivado dos dados da pesquisa de campo^(11)^. Este estudo foi orientado pela pergunta de investigação: Como acontece o automanejo individual e familiar da saúde geniturinária por homens hospitalizados? O desenho do estudo seguiu as diretrizes do COREQ.

### Local

A pesquisa foi realizada em uma enfermaria de urologia de um hospital público universitário da rede federal na Bahia, Brasil. A unidade de referência contempla 23 leitos, alocados em 12 quartos, com atendimento de pacientes de ambos os sexos e identidade de gênero - referência para cirurgias do Processo Transexualizado, com demandas geniturinárias e ginecológicas. A enfermaria presta serviço de atendimento de enfermagem, medicina, nutrição, fisioterapia, bioimagem e laboratório. As principais doenças e agravos encontrados foram: Hiperplasia de próstata, neoplasias malignas de bexiga, pênis, próstata e litíase renal.

### População e Critérios de Seleção

Os participantes do estudo foram homens adultos/idosos internados no hospital com necessidades geniturinárias afetadas, familiares/acompanhantes e profissionais do campo da Enfermagem. Foram atribuídos os seguintes critérios de seleção:

- Homens acima de 18 anos de idade, com demandas de saúde geniturinária, atendidos na unidade hospitalar de urologia. Critérios de exclusão: Homens recém-admitidos na unidade de estudo, apresentar instabilidade hemodinâmica que impossibilite a realização da entrevista;
- Familiares/acompanhantes - ser membro familiar e/ou cuidador durante o internamento. Critérios de exclusão: estar na condição de visitante;
- Profissionais de enfermagem - atuar na enfermaria de urologia investigada. Critérios de exclusão: encontrar-se no período de férias ou em afastamento das funções ocupacionais.

Houve uma recusa expressa de um paciente em decorrência de manifestação dolorosa, de dois acompanhantes, por não sentirem confortáveis para realizar a entrevista e de três profissionais decorrentes da escassez de tempo.

### Coleta de Dados

A coleta de dados iniciou-se no período de julho a setembro de 2024, através de uma equipe de pesquisa composta por estudantes de graduação, mestrado e doutorado e pesquisadores doutores com experiência na temática/metodologia, mediante treinamentos em coleta de dados, sem vínculo direto com os participantes. por meio de observação participante^(12)^, guiada por um instrumento próprio, contendo os tópicos: 1. Características de funcionamento do serviço; 2. Perfil e demandas da clientela (atitudes, comportamentos e práticas de automanejo da saúde geniturinária); 3. Atuação/prática profissional da equipe da Enfermagem; 4. Interprofissionalidade, validado internamente no grupo de pesquisa e submetido à piloto no primeiro dia em campo durante três meses. Buscou-se estabelecer relações com os atores sociais observados, obter compreensão aprofundada do fenômeno e as intervenções de enfermagem. Foram preenchidos 21 roteiros de observação de campo.

Em segundo momento, realizaram-se as entrevistas individuais, em encontros únicos, com tempo médio de duração 50 minutos, num total de 31 horas de material em áudio junto aos pacientes/familiares e profissionais do campo da Enfermagem. Para tanto, empregou-se um instrumento semiestruturado, validado internamente por membros do grupo de pesquisa e submetido à piloto junto a um grupo de cinco participantes. O instrumento foi composto por questões fechadas relacionadas às características sociodemográficas, laborais e de situação de saúde (homens adultos em internação hospitalar), bem como de perguntas abertas relacionadas ao objeto investigado, a saber: Homens - Fale-nos como o Sr. gerencia o cuidado genital e urinário da sua própria saúde; Familiares - Descreva-me como ocorre a sua participação na gestão do cuidado da saúde do homem que está acompanhando; Profissionais - Conte-me como você intervém diante da gestão da saúde geniturinária de homens e a relação com os seus familiares.

As entrevistas foram encerradas quando foi observado o alcance de critérios metodológicos da Análise Temática Reflexiva, a saber: reconhecimento dos significados gerados a partir da interpretação dos dados coletados^(13)^. O material coletado gravado foi submetido à transcrição na íntegra, codificadas manualmente, sendo organizadas, sistematizadas, identificadas em arquivo único sob o auxílio do *Google Drive* ^®^ e planilha em *Excel* ^®^, para a constituição do *corpus* de análise. Verificou-se a integridade, duplicidade e incompletude dos dados.

### Análise e Tratamento dos Dados

Adotou-se o método da Análise de Conteúdo Temática Reflexiva, executada em seis fases: (1) Familiarização com os dados - imersão por meio da leitura e releitura dos dados coletados visando a identificação de ideias iniciais e a extração de rótulos (157); (2) Geração de códigos iniciais - produção sistemática de códigos iniciais (58); (3) Busca por temas, agrupou-se os códigos em possíveis temas (56); (4) Revisão dos temas, refinamento dos temas identificados, verificando a representatividade e coerência interna; (5) Definição e nomeação dos temas, procedeu-se com o refinamento dos temas, atribuindo nomes e descrições claras; (6) Produção do relatório, elaboração do artigo científico^(14,15)^. Adotaram-se passos para alcance da fidedignidade na pesquisa qualitativa: validação interna pesquisa equipe e membros do grupo de pesquisa, apreciação externa de *experts* na área e junto aos participantes^(16)^.

A Teoria do Automanejo Individual e Familiar foi empregada para a interpretação dos achados, a qual explica o automanejo como um fenômeno multidimensional e complexo que afeta indivíduos e familiares. O automanejo tem sido utilizado para se referir a três fenômenos: o processo, relacionado com habilidades de autorregulação para gerenciar as condições crônicas ou fatores de risco, como definição de metas, automonitoramento e como também pensamento reflexivo. O segundo fenômeno: programa/intervenções, o qual se relaciona com a capacitação de pessoas para assumir responsabilidade de gerenciar suas próprias doenças como também se envolver em atividades de promoção, prevenção e recuperação da saúde. O terceiro e último fenômeno, são os resultados alcançados após envolvimento do indivíduo nas ações de automanejo, relacionado ao processo melhora ou estabilização da condição de saúde^(8)^.

### Aspectos Éticos

O projeto foi aprovado sob Parecer CAAE: 76017423.3.3001.0049 e n°: 6.821.769/2024, em acordo com as Resoluções 466/2012, 510/2016 e 580/2018 do Conselho Nacional de Saúde. Aplicou-se um Termo de Consentimento Livre e Esclarecido para a anuência de cada grupo de participantes. Para garantir o anonimato dos participantes foi utilizada a identificação por meio de siglas: H de homem, F de familiar e PE de Profissional do campo da Enfermagem, seguido da categoria exercida, seguido do número de ordem da entrevista: H01, F05 e PE - Enfermeira.

## RESULTADOS

### Caracterização dos Participantes

O estudo foi composto por 19 homens internados, oito familiares/acompanhantes, e 8 profissionais do campo da Enfermagem, totalizando 35 participantes do estudo.

Homens adultos e idosos hospitalizados (19): idade média de 59 anos, em sua maioria de raça/cor autodeclarada preta(11), estado civil casados (12), identidade de gênero cisgêneco (19) orientação sexual heterosexual (19), nível de escolaridade - ensino médio completo (6), tendo apenas um participante com ensino superior completo. Quanto à situação no mercado de trabalho, a maioria eram aposentados (14), sendo que 2 deles eram lavradores. No que tange a renda salarial média, referiram “ter renda e contribuir para o sustento da família” (14). Residem em casa própria (18), todos eles com acesso à energia elétrica e banheiro, contudo, 15 referiram ter água encanada e 13 acesso a saneamento básico, convivendo em área urbana (13) e área rural (6), na presença de suas companheiras afetivo-conjugais (16). A maioria possuía acesso à *Internet* (14), aparelho celular (16).

Familiares/acompanhantes (8): mulheres, sendo 6 esposas, 2 filhas, de raça/cor preta (5), heterossexuais (8), cisgênero (8), sem ocupação (3), seguido de aposentadas (2). Profissionais do campo da Enfermagem (8): sexo feminino (7), raça/cor preta (5), cisgênero (8), heterossexuais (8), formação superior (6) - Enfermeira e formação técnica (2), presença de pós-graduação (5), contudo, nenhuma na área de Enfermagem em Urologia/Nefrologia. Cinco destes possuíam apenas um único vínculo empregatício, com uma trajetória profissional de mais de 10 anos de formação (7), sem experiência profissional prévia na área de Urologia, atuando numa jornada de trabalho semanal de 36 horas.

### Achados Empíricos do Fenômeno Investigado

Os resultados empíricos foram apresentados sob o enquadramento teórico, considerando os aspectos: Elementos constitutivos da Teoria do Automanejo; Substrato da codificação dos dados empíricos e da evidência dos achados empíricos, no que tange: contexto do automanejo; processos de autogestão; resultados proximais e distais e as intervenções aparentes.

### Dimensão Individual do Automanejo

#### Tema Gerador Inicial: O Contexto do Automanejo da Saúde Geniturinária

O contexto do automanejo da saúde geniturinária desempenhado por homens adultos em hospitalização atrela-se à cuidados básicos de higiene íntima, na justificativa da ausência de agravos genitais, histórico familiar de doenças e agravos geniturinários, especialmente, entre outros homens da família, a manutenção de vínculo com ambiente considerado saudável, a evolução de piora da condição clínica, levando-os à intervenções, procedimentos e a hospitalização e percepção de apoio por parte dos membros da família.

Os familiares estiveram envolvidos num contexto em que o automanejo da saúde geniturinária dos homens hospitalizados demanda ter que migrar de cidade, percorrer itinerários terapêuticos no centro urbano, em unidade hospitalar de referência, tendo vivenciado o acesso a outros serviços de saúde disponíveis na rede de atenção na condição de acompanhantes, sendo expressivamente composta por mulheres, quer sejam as companheiras afetivo-conjugais, como filhas e outras mulheres do ciclo familiar. Tais mulheres buscam cooperar com a promoção de um ambiente familiar saudável, incluindo a enfermeira hospitalar, o suporte às medidas de tratamento urológico empregadas pela equipe de saúde, bem como de cuidados com a alimentação, o uso de medicamentos prescritos. Além disso, expressaram a percepção acerca da pessoa significativa em relação com a saúde e a vivência de dificuldades para desempenhar a função de acompanhante. Desse modo segue o Quadro 1 com o enquadramento teórico de contexto do automanejo individual e familiar.

**Quadro 1:** Enquadramento teórico do contexto do automanejo individual e familiar da saúde geniturinária de homens hospitalizados – Salvador, BA, Brasil, 2025.

Elementos constitutivos da Teoria do Automanejo	Substrato da codificação dos dados empíricos	Achados empíricos
**Contexto:** Fatores protetores: noção de higiene íntima; correlação do uso do sabonete para a higiene íntima; ambiente rural, doméstico e laboral; presença e prontidão dos familiares; Riscos: não avanço nos cuidados de higiene íntima em razão da ausência de doença/agravo; presença e recorrência de infecções urinárias; histórico de doença geniturinária na família - câncer de próstata.		
**Condição – fatores específicos; percepção individual/familiar** (Complexidade da condição e tratamento, trajetória, estabilidade de condição e transições)	Tema: A higiene íntima com o uso de sabonete comum; Tema: Cuidados básicos de higiene íntima em razão da ausência de problemas genitais; Tema: Lavagem e uso de produtos na higienização do pênis; Tema: Historicidade de eventos geniturinários ao longo da vida; Tema: Progressividade do problema de saúde geniturinária; Tema: Submissão à procedimentos para solucionar problemas urinários; Tema: Multiplicidade de doenças e agravos geniturinários; Tema: Vivência de eventos críticos de saúde geniturinária; Tema: Experiência de complicações da função urinária/miccional; Tema: Trajetória percorrida envolvendo a migração interestadual; Tema: Acompanhamento familiar na transferência para o hospital; Tema: Acompanhamento familiar em serviço ambulatorial especializado	[...] *eu não utilizo nenhum produto de higiene específico. Realizo cuidados comuns, como o uso de sabonete, até porque também não tenho problema genital algum. Nunca tive doenças antes.* (H01);[...] *meu problema de saúde já vem de muitos anos, eu fui operado há muitos anos atrás, e até hoje continuo tendo muitas crises. Já tive muitas infecções urinárias e fui pra máquina para “quebrar as pedras”* (refere-se à realização do procedimento de litotrícia). (H05); [...] *tem dias que vou quatro a cinco vezes ao banheiro para urinar. O problema é que a urina fica pingando quando faço xixi.* (H07); [...] *o que me afetava era ter que ir várias vezes ao banheiro para urinar.* (H08); [...] *nós fomos encaminhados pelo serviço do município que moramos e aí chegamos até aqui* (refere-se ao hospital de referência no estado)*.* (F05); [...] *ele teve acesso à policlínica da minha região onde viva.* (F05).
**Ambiente físico e social** (Acesso à saúde, transporte, cultura, capital social)	Tema: Ampliação do acesso ao serviço de saúde urológica; Tema: Enfrentamento de dificuldades na assistência hospitalar em urologia; Tema: Manutenção da convivência em ambiente rural; Tema: Manutenção das atividades laborais; Tema: Promoção de ambiente saudável pelo familiar; Tema: Suporte familiar no tratamento urológico.	[...] *o hospital necessita abrir as portas para que nós homens não tenhamos dificuldade, evitando que eles desistam de se cuidar pelo fato de enfrentar dificuldade para conseguir uma consulta, agendar o procedimento, a vaga no hospital e o transporte para o deslocamento.* (H01); [...] *eu penso em manter convivendo na roça, realizando o meu trabalho,* me *alimentando bem para viver mais.* (H03); [...] *cuido da alimentação, da casa, sempre procuro manter o ambiente limpo, saudável, higienizável, as roupas limpas.* (F02); [...] *quando surgiu o problema de saúde nós começamos a buscar tratamento na cidade mais próxima, mas não conseguimos resolver, tivemos que viajar e ser encaminhado para o hospital na capital.* (F05).
**Fatores individuais e familiares** (Estágios de desenvolvimento, capacidade de aprendizagem, alfabetização, estrutura e funcionamento familiar, capacidade de autogestão)	Tema: Noção de comprometimento da saúde geniturinária mediante às Infecções Sexualmente Transmissíveis; Tema: Percepção de que os homens estabelecem multiplicidade de parcerias sexuais; Tema: Conhecimento limitado das doenças e agravos geniturinários; Tema: Prática da alimentação saudável como estratégia de manejo da função geniturinária; Tema: Emprego de medidas de higiene geniturinária e percepção de alcance de bons resultados; Tema: Atribuição de relações causais de complicações urinárias - cálculos renais; Tema: História de enfermidade renal e geniturinária entre homens da família; Tema: Mortalidade paterna e a relação com o câncer de próstata; Tema: Apoio familiar e protagonismo das companheiras afetivo-conjugais; Tema: Suporte familiar na prestação de cuidados de alimentação e medicalização; Tema: Percepção familiar da condição de saúde da pessoa significativa; Tema: Vivenciando dificuldades na apreensão de conhecimento para cuidar do familiar.	[...] *eu sei que algumas doenças transmissíveis podem causar problemas nos órgãos genitais e urinários, como o cancro, no caso dos homens que têm muitos parceiros sexuais.* (H01); [...] *já ouvi falar sobre infecção urinária* [...] *meu pai tinha infecção urinária.* (H03); [...] *procuro não me alimentar com alimentos gordurosos, não pegar peso. Além disso, tomo cuidados com a sonda vesical, evitando tocá-la no chão, deixando-a pendurada para evitar que as bactérias cheguem até a bexiga.* (H04); [...] *lavo bem o pênis e realizo a limpeza após urinar* [...] *as pessoas comentam que cálculo renal é devido a pouco líquido e eu bebo bastante água* [...] *tenho dois irmãos utilizando dreno no rim.* (H05); [...] *eu preciso me cuidar, procurar ser limpo* [...] *meu pai teve antes de morrer a doença da próstata, a próstata ficou alterada ao ponto de não conseguir urinar.* (H06); [...] *tenho apoio da família. A minha esposa se prontificou e teve boa vontade para ficar comigo e cuidar de mim. Nós temos uma boa convivência, apesar do pouco tempo juntos.* (H07); [...] *acho que faço corretamente a minha higiene íntima.* (H08); [...] *bebo pouca água, mas vou beber mais. Utilizo sabão para higiene íntima* [...] *dois irmãos tiveram problema na próstata*. (H09); [...] *eu tenho uma família grande e por esse motivo eu tenho sempre com quem contar para me apoiar no cuidado com a minha saúde, principalmente agora, que estou internado e precisei realizar uma cirurgia.* (H10); [...] *eu que dou os medicamentos, eu que faço a alimentação dele, mas banho e outras questões ele consegue fazer sozinho.* (F03); [...] *não tinha conhecimento do problema de saúde dele.* Só *fiquei sabendo do depois da internação.* (F06); [...] *ele está com diagnóstico de doença renal e da próstata, mas na próstata não precisa de intervenção agora. Já ouvi falar de doença renal, mas confesso que não tenho conhecimento o suficiente para cuidá-lo.* (F07).

O processo de autogestão da saúde geniturinária empregado por homens adultos hospitalizados expressou (Figura 1), conhecimentos e crenças relacionadas as razões geradoras de enfermidades geniturinárias, com ênfase às Infecções Sexualmente Transmissíveis, o uso de fitoterápicos, muitas vezes, indicados por pessoas próximas, de uso por conta própria e sem indicação de profissional da saúde.

**Figura 1:** Modelo explicativo da aplicação da Teoria do Automanejo Individual e Familiar da saúde geniturinária de homens adultos hospitalizados. Salvador, BA, Brasil.

Fonte: Adaptado de Ryan, Sawin, 2009.

Emergiram metas estabelecidas para o momento atual da internação e futuro, numa perspectiva de melhoria da atividade física, alimentação, uso de medicamentos e autocuidado. Além disso, manifestou mecanismos de autocontrole dos sintomas e queixas geniturinárias como a autoinspeção e a vigilância quanto à eliminação urinária. Trouxe à tona ainda, a deflagração de pensamentos reflexivos acerca da sua condição de saúde atual e das possibilidades de ressignificação de hábitos e práticas de cuidado com a saúde geniturinária, graduando e/ou nivelando o seu estado de saúde. Para o suporte ao automanejo, os familiares fizeram referência à Deus, expressando percepções acerca da relação entre o familiar e o cuidado da sua saúde, permeada por contexto de agravamento da condição de saúde geniturinária e o surgimento de complicações, atreladas às estratégias de apoio que são concedidas aos homens. Outrossim, os familiares tiveram que lidar com a apreensão da cirurgia, a busca pela facilitação do automanejo, na medida em que expressaram afeição pelo familiar enfermo, demonstrando dedicação e atribuindo valor ao cuidar do outro, a maioria, companheiros afetivo-conjugais. Desse modo segue o Quadro 2 com o enquadramento teórico do processo de autogestão do automanejo individual e familiar.

**Quadro 2:** Enquadramento teórico do processo de autogestão do automanejo individual e familiar da saúde geniturinária de homens hospitalizados – Salvador, BA, Brasil, 2025.

Elementos constitutivos da Teoria do Automanejo	Substrato da codificação dos dados empíricos	Achados empíricos
**Processo de autogestão**		
**Conhecimentos e crenças** (Informações factuais, autoeficácia, expectativa de resultado, congruência de metas)	Tema: Atribuindo relações das masculinidades à saúde geniturinária; Tema: Aderindo aos fitoterápicos sob a indicação de pessoas próximas; Tema: Assumindo comportamento de evitação para evitar complicações da saúde geniturinária; Tema: Concepção de saúde para além da doença e a busca ampliada de profissionais médicos de especialidades diversas; Tema: Atribuição de faixa etária enquanto marco para estabelecer medidas de automanejo da saúde-doença; Tema: Busca por profissional médico urologista; Tema: Referência à Deus como benéfico e promotor de conforto, demonstrando serví-lo.	[...] *existem muitos homens que não usam camisinha no sexo, faz tudo o que deseja. Por esse motivo surgem os problemas como o HTLV, HIV, Sífilis e tudo isso compromete a função genital e urinária.* (H02); [...] *fazer chá de quebra pedra, mandacaru, ou outro que as pessoas me indicam.* (H05); [...] *estou procurando correr atrás da minha saúde para não deixar os problemas acumularem, se não será bem pior, não posso parar.* (H07); [...] *saúde é prevenção, preciso me cuidar mesmo sem estar doente, ir ao médico, não só ao urologista como também outras especialidades.* (H08); [...] *faço os cuidados normais. Se for necessário utilizar o medicamento eu utilizo;* (H10); [..] *a partir dos 40, 45 anos o homem tem que procurar o urologista* [...] *desde que adoeci eu procurei ser servo de Deus, tive problemas do passado que me prejudicava e não me deixavam bem. Foi quando ouvi a palavra de Deus, serviu de conforto.* (H11). [...] *graças a Deus eu fiquei bem ao vê-lo retornar da cirurgia* [...] *vou continuar pedindo a Deus que conceda saúde a ele.* (F05).
**Competências e habilidades de autorregulação** (Estabelecimento de metas, automonitoramento e pensamento reflexivo, tomada de decisão, planejamento e ação, autoavaliação, controle emocional)	Tema: Busca pela longevidade a partir da alimentação saudável, atividade física e de lazer; Tema: Aspirando reflexões sobre si mesmo e a sua condição de saúde atual e projeções futuras; Tema: Cumprimento de terapêutica médica relacionada à medicalização; Tema: Atribuindo parâmetros de autoavaliação da saúde; Tema: Adotando comportamentos de alerta para o consumo de água; Tema: Identificando sintomas urinários desagradáveis; Tema: Inspeção dos órgãos genitais com menção ao pênis e os testículos; Tema: Explicitando características comportamentais e traços de personalidade relacionados aos cuidados da saúde; Tema: Manifestando tolerância à dor e outros sintomas; Tema: Revelando a percepção da relação do familiar com a saúde; Tema: Revelando a gravidade do problema de saúde geniturinária do familiar e as estratégias de apoio concedidas.	[...] *busco realizar exercícios físicos, ler, ver o que é melhor.* (H01); [...] *se eu não cuidar de mim mesmo quem vai cuidar?.* (H06); [...] *eu me alimento bem, faço uso das medicações, faço o que os médicos pedem* [...] *se eu não me cuidar agora, e amanhã? Hoje estou com 90% do fluxo urinário. Se deixar para cuidar amanhã, como vou estar?* (H04); [...] *estou me cuidando, hoje em dia precisamos nos cuidar, o médico mandou.* [...] *fico em alerta para tomar bastante água. O que mais costumo fazer agora é seguir com a orientação de beber bastante líquido. Sinto mau cheiro na urina, quando não bebo muita água.* (H05); [...] *eu olho como estão os meus testículos.* (H07); [...] *os parentes da minha família costumam chegar aos 100 anos e eu também pretendo chegar até essa idade sem precisar do auxílio de ninguém.* (H10); [...] às vezes sou teimoso, demoro para ir ao serviço de saúde [...] *sou um pouco cabeça dura, senti dor e ficava muito tempo em casa resistindo, não era muito disciplinado, mas agora preciso manter a disciplina* [...] *com tudo que tem acontecido comigo, vai abrindo meus olhos, meu corpo deu sinal, o corpo nos dá sinais, não é? Eu ficava muito tempo em casa questionando: será que vai passar essa dor? Agora eu estou fazendo uma reflexão de mim mesmo, da minha saúde, vamos dizer assim... saúde boa é quando está 100%. A minha eu vejo que está em 40%.* (H11); [...] *ele tem dificuldade de ir ao médico, não gosta. Quando a família descobriu que ele estava com esse problema, logo começamos a buscar meios, ir a procura de um médico.* (HA05).
**Facilitação social** (Influência social, apoio – emocional, instrumental ou informativo, colaboração negociada)	Tema: Acompanhamento familiar presente no internamento hospitalar urológico; Tema: Protagonismos das esposas enquanto acompanhantes na unidade hospitalar; Tema: Apreensão de informações em saúde repassadas por profissionais médicos; Tema: Entre a apreensão da cirurgia e o alívio em ter o familiar por perto; Tema: Dedicação familiar à prestação de cuidados; Tema: Atribuindo valor ao cuidado prestado ao familiar esposo.	[...] *minha família sempre me acompanhou. Minha esposa me acompanhou até aqui no internamento hospitalar.* (H07); [...] *sobre as doenças como o câncer, e a infecção urinária eu sempre vejo os médicos falando.* (H11); [...] *eu me senti nervosa na hora da cirurgia dele, mas tudo foi um sucesso e hoje ele está bem, internado, recebendo o meu apoio.* (HA05); [...] *dedico todas as horas, cuido dele o dia todo, todos os dias, como na alimentação, roupa e outras coisas, lembro dos remédios e acompanho ao médico.* (HA06); [...] é meu esposo, eu tenho que cuidar, acompanhar da melhor maneira possível, e da melhor forma que eu posso. (HA07).

Os resultados que emergiram do automanejo da saúde geniturinária empregado por homens adultos hospitalizados revelaram numa dimensão proximal, a busca por assistência médica à saúde, com presença da companhia afetivo-conjugal, aparição da figura da enfermeira enquanto promotora da educação em saúde, acesso à serviços de saúde de urgência e emergência, adesão à Práticas Integrativas e Complementares ligadas aos fitoterápicos. Diante ao contexto dos resultados distais obtidos na experiência do automanejo da saúde geniturinária, os familiares expressaram afinidade para cuidar e o sentimento de bem-estar em desempenhar esse exercício, que se entrelaça com a manifestação do cansaço, sobrecarga e preocupações para com o familiar hospitalizado (Quadro 3), o que suscita intervenções de enfermagem, as quais estiveram concentradas no manejo clínico e na execução de procedimentos de enfermagem, com limitada intervenção educativa e promotora do automanejo individual e familiar (Quadro 4).

**Quadro 3:** Enquadramento teórico dos resultados proximais e distais do automanejo individual e familiar da saúde geniturinária de homens hospitalizados – Salvador, BA, Brasil, 2025.

Elementos constitutivos da Teoria do Automanejo	Substrato da codificação dos dados empíricos	Achados empíricos
**Resultados proximais e distais**		
**Resultados proximais – comportamentos de autogestão individual e familiar** (Envolvimento em atividades/regimes de tratamento, uso de terapias farmacológicas, gestão de sintomas)	Tema: Acompanhamento médico enquanto atividade/regime de tratamento geniturinário; Tema: Presença feminina enquanto promotora da saúde dos homens com enfermidades geniturinárias; Tema: Enfermeira enquanto profissional que ensina, orienta e dá treinamentos; Tema: Automedicação e uso de fitoterápicos como práticas; Tema: Busca por profissional médico em situação secundária as tentativas de automanejo em domicílio; Tema: Busca por serviços de urgência e emergência; Tema: Profissional médico enquanto ponto focal de primeira procura; Tema: Uso de medicamentos como recurso terapêutico de gestão dos sintomas.	[...] *no meu caso foi acompanhamento médico eu e minha esposa. Busco assistência médica.* (H01); [...] *tenho sempre procurado os médicos para me orientar. É sempre bom ter orientações através dos médicos* [...] *a enfermeira me ensina e orienta para que eu faça os treinamentos aqui no hospital.* (H02); [...] *tomava remédio por conta própria, usava uma planta e fazia o meu chá. Remédio não. Tirava uma folha de boldo, cozinhava e tomava para aliviar, quando a situação apertava procurava um médico.* (H03); [...] *busca me cuidar pois o médico mandou*. (H04); [...] *consumo líquido, chá da folha de quebra pedra e cana do macaco do brejo* [...] *quando não resolve eu procuro o médico.* (H05); [...] buscava procurar um médico, e isso ocorria na UPA*. Tomo remédio como dipirona e chá de boldo, capim santo, capim cidreira, quando estou em casa. (H06); [...] *fui direto ao médico na clínica.* (H07); [...] *já necessitei usar medicamentos por conta própria.* (H10). ^*^ Unidade de Pronto Atendimento 24 horas.
**Resultados proximais – custo dos serviços de saúde** (O custo associado ao uso de cuidados de saúde)	Tema: Adoção de medidas consideradas saudáveis diante da impossibilidade de arcar com outros recursos; Tema: Necessidade de assistência farmacêutica. Tema: Desembolso de recurso financeiro com seguradora de saúde.	[...] *hoje eu procuro a melhor maneira possível de viver a vida saudável, pois não tenho como arcar com um plano de saúde.* (H09); [...] *já necessitei fazer uso de medicamentos e hoje eu pago por um plano de saúde*. (H10).
**Resultados distais – estado de saúde** (Prevenção, atenuação, estabilização)	Tema: Estabelecimento de cuidados genitais preventivos; Tema: Adoção de cuidados de higiene com dispositivo urinário; Tema: Emprego de fitoterápicos sob o consumo de chás enquanto solução de alívio de sintomas; Tema: Busca por assistência médica com atribuição de periodicidade; Tema: Prática da automedicação enquanto recurso terapêutico.	[...] *estou bem com a saúde, pois mantenho a higiene, o pênis está sempre limpo e a sonda vesical também.* (H04); [...] *o problema do meu rim a salvação foi fazer uso do chá. Quando eu tomava chá, resolvia e me fazia sentir bem.* (H06); [...] *busco o médico, atualmente, mas antigamente quando eu tinha uma dor, procurava tomar um remédio.* (H09).
**Resultados distais – qualidade de vida** (Bem-estar percebido, custo da saúde)	Tema: Satisfação na busca pela melhoria da condição de saúde e de obter ajuda do outro; Tema: Percepção de bem-estar ao cuidar de si da saúde; Tema: Sentimento de gratidão atrelado ao vínculo familiar; Tema: Sentimento de otimismo e utilidade; Tema: Sentimento de realização atrelado à coesão familiar; Tema: Percepção de priorização de si da saúde; Tema: Afinidade para cuidar e sentimento de bem-estar no manejo da saúde-doença do familiar.	[...] *estou satisfeito buscando a melhora. Vou amadurecendo e buscando o melhor pra mim.* (H01); [...] *me sinto satisfeito de ter pessoas me ajudando.* (H03); [...] *me sinto bem, me faz bem e eu preciso me cuidar para o meu bem-estar.* (H05); [...] *os chás que eu tomava me faziam sentir bem. Me sinto bem cuidando da minha saúde. Tenho gratidão pela família que tenho e que me faz sentir bem.* (H06); [...] é ótimo cuidar de mim mesmo e saber que sou útil. (H09); [...] *me sinto realizado com o apoio familiar que tenho para cuidar da minha saúde.* (H10); [...] *eu me sinto bem pois estou cuidando da minha saúde, que agora é uma prioridade.* (H11); [...] *quando eu cuido é porque eu gosto de vê-lo se sentindo bem e isso me faz me sentir bem também.* (HA02); [...] *eu me sinto bem pois convivo bem com ele, graças a Deus, é um ótimo esposo, então, eu tenho que cuidar.* (A05).
**Resultados distais – custo da saúde** (Custo direto e indireto)	Tema: Cancelamento de medidas para manter os cuidados com a saúde; Tema: Desembolso de recursos financeiros em prol da manutenção da saúde; Tema: Cansaço, sobrecarga e preocupações experienciada pelo familiar.	[...] *eu tinha plano de saúde, mas como estava caro eu cancelei*. (H09); [...] *eu pago um plano de saúde para vir me visitar*. (H10); [...] é um cansativo, pois o meu pai precisa de cuidados o tempo todo. *Ficamos preocupadas com o diagnóstico e a situação de saúde dele. E particularmente, fiquei ansiosa em acompanhá-lo.* (HA03); [...] *tenho que fazer por ele, às vezes me sinto muito cansada, sobrecarregada. A cirurgia demorou muito e fiquei preocupada.* (A04); [...] *fiquei com medo, e quanto mais passava o tempo de retornar do centro cirúrgico eu entrava em desespero. Retornou no período da noite* (A05).

#### Dimensão Profissional do Automanejo - Profissionais do Campo da Enfermagem

**Quadro 4:** Enquadramento teórico das intervenções com o enfoque no automanejo individual e familiar da saúde geniturinária de homens hospitalizados – Salvador, BA, Brasil, 2025.

Elementos constitutivos da Teoria do Automanejo	Substrato da codificação dos dados empíricos	Achados empíricos
**Intervenções**		
**Intervenções centradas no indivíduo e família**	Tema: Execução de técnicas e procedimentos de enfermagem; Tema: Controle da eliminação urinária por meio da irrigação vesical; Tema: Desobstrução da sonda; Tema: Exame físico do aparelho genital; Tema: Controle do balanço hídrico; Tema: Controle de episódios hemorrágicos; Tema: Estabelecimento de diálogo para ampliação do conhecimento em saúde pelos homens pacientes.	[...] *nas medicações, trocas de aparelhos e dispositivos.* (P01); [...] *realizando curativos para feridas infectadas, utilizando produtos como carvão ativado alginato* [...] *irrigação vesical, controle de hemorragias, registros de enfermagem.* (P02); [...] *administração de medicação, cuidados com os drenos, eliminação das secreções, irrigação vesical.* (P03); [...] *controlar de forma efetiva o balanço hídrico, as irrigações vesicais.* (P04); [...] *tento conversar com os pacientes para que tenham conhecimento da sua condição de saúde.* (P05); [...] *realizo a desobstrução da sonda vesical, principalmente, quando o paciente retorna do centro cirúrgico* [...] *exame físico do genital.* (P05).

## DISCUSSÃO

Os resultados deste estudo revelaram uma predominância de indivíduos negros e pardos, cisgêneros, heterossexuais, casados, com diferentes níveis de escolaridade e em sua maioria aposentados, com média de 59 anos. Considerar o perfil dos homens entrevistados é primordial para analisarmos o automanejo individual e familiar da saúde geniturinária destes, uma vez tais características podem influenciar no processo de cuidado.

No que diz respeito ao contexto do automanejo da saúde geniturinária, entre os fatores protetores e de risco, se destacaram os cuidados básicos com a higiene íntima, histórico familiar de doenças e agravos geniturinários, a figura feminina como promotora de cuidado, com enfoque nas esposas, a manutenção com o ambiente considerado saudável, o não avanço na higiene íntima atrelado a ausência de doença surgiram como fatores risco. Os fatores contextuais explicitados na Teoria contribuem para a compreensão ampliada de como o automanejo é desempenhado no âmbito individual e na díade intrafamiliar ou na unidade familiar. Tais fatores contextuais podem afetar o automanejo, comprometer o processo, diante as dimensões contextuais e processuais existentes na gestão da saúde, especialmente, no viver com a funcionalidade geniturinária afetada. Quando as intervenções de enfermagem consideram o contexto, podem contribuir para a redução de riscos em saúde e promover condições favoráveis ao automanejo, podendo ser evidenciado em amplitude nos nossos achados^(8)^.

No que concerne à higiene íntima amplamente mencionada, achados na literatura apontam que saberes masculinos associados ao cuidado com o corpo, frequentemente estão associados à noção de higienização corporal, com relevante importância aos órgãos genitais, associada a inspeção contínua desses^(17)^. Contudo, é importante destacar que os cuidados geniturinários não devem ser limitados apenas aos órgãos genitais, sendo necessários cuidados mais amplos e integrados à saúde geral.

Neste estudo foi evidente a ausência de homens como cuidadores, em contrapartida a presença feminina surge enquanto promotora da saúde dos homens e protagonista do automanejo familiar, o que pode ser correlacionado com questões socioculturais, no qual o cuidar se apresenta com escasso reconhecimento social e forte feminização^(18)^. As mulheres, frequentemente esposas e filhas, são co- responsáveis pela gestão dos cuidados, principalmente na presença de um ente adoecido, sendo permeadas por sentimentos que envolvem bem estar por cuidar e manejar a saúde-doença do familiar, mas também por responsabilidade moral, obrigação, além de dificuldades com a sobrecarga, cansaço e preocupações para com o familiar hospitalizado^(18)^.

No processo de autogestão da saúde geniturinária foram evidenciados a influência de conhecimentos e crenças, com uso constante de fitoterápicos, automedicação e o estabelecimento de metas numa perspectiva de melhoria. Os achados do presente estudo apresentam os componentes de processo do automanejo notórios, o que podem apoiar na compreensão dos resultados, quer sejam eles proximais ou distais do manejo da saúde geniturinária^(8)^. O uso de fitoterápicos, muitas vezes indicado por vizinhos, amigos e familiares, apresenta nuances complexas. O uso de plantas medicinais para promover saúde e/ou complementar tratamentos de determinadas condições deve ser visto com cautela, uma vez que seu uso não é isento de riscos^(19)^. Tais achados podem ser explicados pela Teoria do Automanejo quando intervenções de enfermagem direcionadas ao processo do automanejo cooperam para o aprimoramento do conhecimento e das crenças em saúde, bem como podem ajudar a aumentar a adoção de comportamentos de autorregulação de si e da saúde pelo indivíduo, facilitando a socialização^(8)^.

Ademais, apesar do Brasil possuir regulamentação por meio da Política Nacional de Plantas Medicinais e Fitoterápicos e incluí-la nas Práticas Integrativas e Complementares do SUS, estudos indicam conhecimento equivocado em vários aspectos importantes, como efeitos adversos desta terapêutica, desconhecimento de substâncias quimicamente ativas, além do desconhecimento das interações medicamentosas^(19,20,21)^. Neste cenário é primordial explorar conhecimentos multi e interdisciplinares envolvendo o conhecimento popular e científico, na busca por estratégias que promovam o bem estar e reduzam ou anulem a possibilidade de danos ao usuário e agravamento de determinada condição^(19)^.

Nos resultados proximais e distais, envidou-se a figura médica como referência, a busca secundária pelo profissional médico, o desembolso de recursos financeiros e dificuldade acesso ao serviço especializado. Faz-se pontuar que na Teoria do Automanejo Individual e Familiar, a influência social é concebida como uma mensagem ou diálogo, nos quais pessoas em posição de autoridade, respeitadas e com conhecimento especializado, aconselham e encorajam comportamentos de saúde^(8)^. Assim, o médico reiteradamente mencionado, enquadra-se neste papel, uma vez que é visto como profissional de primeira escolha e como impulsionador de cuidados, no qual alguns pacientes, realizam determinadas práticas de saúde unicamente porque foram orientados pelo médico, o que pode ser reflexo de uma assistência à saúde médico-centrada. Outrossim, reflete a importância da relação médico-paciente, dado seu papel crítico na prevenção, diagnóstico e tratamento da doença, bem como no sucesso terapêutico^(19,22)^.

No que se refere a busca pelos serviços de saúde, existe uma relação íntima com os padrões tradicionais de masculinidade. As normas socioculturais desempenham um papel significativo na relutância dos homens em procurar os serviços de saúde, muitas vezes pelo receio de parecer fraqueza, pelo estigma e descriminação, além da escassez de profissionais masculinos^(23,24)^. Destaca-se ainda a presença do idadismo nos discursos, no qual é citado uma delimitação de faixa etária enquanto marco para estabelecer medidas de automanejo da saúde-doença. Faz-se pontuar que esse fenômeno pode ser influenciado pela idade, gênero, escolaridade e medo da morte^(25)^.

O acesso aos serviços de saúde é determinante para o alcance terapêutico. Neste estudo foi evidenciado obstáculos que impactam neste acesso, tais como itinerários terapêuticos longínquos percorridos pelos homens e seus familiares que residem em municípios distantes da capital, barreiras no acesso aos serviços diante da complexidade em agendar consultas e procedimentos, junto a disponibilidade de vaga no hospital. Corroborando comestes achados, um estudo que analisou as barreiras de acesso em cinco regiões do Brasil, evidenciou dificuldades referentes à acessibilidades geográfica, associadas à indisponibilidade de consultas especializadas na própria Região de Saúde, insuficiência de vagas de internação, tempo de espera prolongado, integração falha ou inexistente dos serviços^(26)^. Soma-se a isto, o fato de que determinados problemas geniturinários como as incontinências, podem gerar impactos multidimensionais, afetando não apenas a qualidade de vida dos pacientes, mas a estrutura, o processo e os resultados assistenciais^(27)^.

Os achados revelam que a dimensão profissional do automanejo no que tange a atuação dos profissionais do campo da Enfermagem estiveram centrados na execução de técnicas e procedimentos assistenciais, sem a aparição de intervenções de enfermagem focadas na educação, aplicação de recursos tecnológicos, como no caso da indicação de aplicativos disponíveis para o manejo de problemas de saúde geniturinárias decorrentes do câncer de próstata, das cirurgias de prostatectomia radical^(28)^. A Teoria do Automanejo pode ser útil para orientar as intervenções de enfermagem individuais e centradas na família, uma vez que impactam positivamente no automanejo da saúde, especialmente, quando consideram o contexto e o processo^(8)^. Assim como de recursos de apoio diagnóstico/terapêutico das incontinências por enfermeiros^(29)^. Logo, o estabelecimento de medidas, programas com o enfoque na autonomia, habilidades, estabelecimento de metas, transição do cuidado, reabilitação, preparo e planejamento para a alta hospitalar, bem como da escassez de intervenções específicas para a promoção e manutenção da saúde geniturinária no contexto hospitalar pesquisado e com implicações no autocuidado^(30)^.

Ressalta-se ainda que as profissionais da enfermagem pesquisadas não apresentaram formação nem especialização na área de Enfermagem em Urologia, o que pode implicar em atuação pouco abrangente e fragilização da assistência de enfermagem no atendimento ampliado das necessidades de saúde geniturinária. É urgente e necessário o emprego de esforços no âmbito da formação em Enfermagem na Saúde dos Homens, da Enfermagem Nefrológica e em Urologia, bem como o fortalecimento dos processos de trabalho em enfermagem, na aplicação do Processo de Enfermagem e das Teorias de Enfermagem, que deem suporte à tomada de decisão clínica das equipes da enfermagem que atuam nesse segmento. Contudo, orienta-se para a cautela na compreensão dos conceitos essenciais e dos construtos das dimensões do automanejo da saúde geniturinária por parte dos homens, considerando que os resultados alcançados pelos homens podem ser facilmente afetados pelas dimensões de contexto e de processo. Isto demandará dos profissionais de enfermagem maior investimento na avaliação clínica de enfermagem (anamnese, exame clínico) – redução de morbimortalidade, controle de taxas metabólicas, prevenção e manejo de condições crônicas, uso preciso de medicamentos, com vista a melhoria do estado de saúde a partir da autogestão^(8)^.

Outrossim, dos profissionais do campo da Enfermagem que atuam em outros pontos da rede de Atenção Primária à Saúde, como corroborado em estudo anterior que evidencia as contribuições da atuação do enfermeiro no tratamento da Disfunção do Trato Urinário Inferior^(29)^. Neste sentido, recomenda-se atentar para o que aponta a Teoria do Automanejo. A mesma indica que, construções baseadas no contexto, processo e resultados combinadas entre si de forma única, podem implicar em avanços substanciais para a prática clínica do automanejo da doença crônica pelos homens acometidos, tornando-os mais engajados a adotarem comportamentos específicos em relação à saúde e a enfermidade geniturinária^(8)^.

Este estudo apresenta potencialidades e implicações para a produção do conhecimento e a sua tradução e para a prática profissional em Enfermagem: analisa e explica o fenômeno do automanejo; apresenta aspectos particulares e singulares de gênero/masculinidades na relação entre cuidado e autogestão da saúde por homens; fortalece os estudos clínico-sociais no contexto da atenção hospitalar; explora a necessidade geniturinária de saúde como foco e agenda de pesquisa; elenca os elementos constitutivos do automanejo, os quais se mostram úteis para direcionar/guiar o raciocínio clínico/diagnóstico do profissional enfermeiro e direcionar a equipe da Enfermagem na produção do cuidado geniturinário da saúde. Além disso, comprovou-se o potencial prático de uma Teoria de Enfermagem de Médio Alcance, a qual mostrou-se adequada à especificidade investigada, podendo ser explorada em investigações futuras e em intervenções assistenciais e educativas^(8)^.

Este estudo apresenta como limitações: a técnica e o método empregado envolveram grupos distintos, os quais podem demandar utilização de recursos diversificados e específicos para a apreender mais aprofundada, explicativa e reflexiva acerca do fenômeno investigado, o que pode limitar a capacidade de abstração, bem como a apreensão dos padrões, propriedades e dimensões do dado encontrado. O fato da pesquisa ter sido realizada no contexto clínico-assistencial hospitalar pode ter reduzido a capacidade de abstração por parte dos profissionais do campo da enfermagem, diante das dispersões, múltiplas atenções e demandas do processo de trabalho, bem como a censura de determinadas informações sensíveis e relevantes a serem levantadas nos participantes homens e em seus familiares/acompanhantes, devendo ser levado em consideração.

## CONCLUSÃO

Os elementos constitutivos do automanejo individual e familiar foram encontrados na experiência de homens com necessidades de saúde geniturinárias afetadas em hospitalização e favorecem o avanço na prática clínica de Enfermagem ao revelar o contexto e o processo em que e como o automanejo ocorre, os resultados que são alcançados na interação com a família e o escopo, a abrangência e os direcionamentos das intervenções de enfermagem que são implementadas, o que explicita indicativos de ampliação, fortalecimento e especificidade. No âmbito da dimensão individual o contexto é marcado pela experiência do internamento hospitalar, com automanejo da saúde geniturinária fragilizado na vida cotidiana. As práticas de cuidado atrelam-se à higiene íntima, assistência à saúde médico-centrada e o incremento de alternativas da fitoterapia automedicalizada. Busca por serviços de saúde de média e alta complexidade diante da piora da condição clínica urológica, tendo a presença feminina como ponto de referência familiar de apoio.

O processo de autogestão sustenta-se em crenças relacionadas à saúde-doença geniturinária, estabelecimento de metas para restabelecer a condição de saúde e o funcionamento geniturinário, permeadas por autoreflexões e autoavaliação da saúde, que se somam às experiências psicoemocionais dos familiares que os acompanham durante o internamento hospitalar. Os resultados proximais e distais indicam a adoção de comportamentos de autogestão, contudo, mobilizados pelo tratamento e terapêuticas medico-centradas, que repercutem em custos para si e para o sistema de saúde, mas que expressam sentidos de melhoria da qualidade de vida por parte dos homens pesquisados.

A atuação familiar é expressiva no contexto do automanejo no que tange aos fatores específicos e a percepção acerca do familiar hospitalizado, cujo funcionamento familiar expressa coesão e suporte, contudo, permeada por dificuldades quanto ao conhecimento acerca do processo saúde-doença geniturinário. Há a crença familiar de que Deus concede conforto, cuja competências e habilidades de autorregulação revelou a resistência dos homens no manejo autônomo da sua saúde e o agravamento dos problemas de saúde geniturinária, o que demanda o emprego de estratégias de apoio aos homens com funcionalidade geniturinária afetada. O resultado do automanejo apoiado pelos familiares, implica em benefícios para si, mas não apontam para as contribuições proximais para os homens.

## DISPONIBILIDADE DE DADOS

Todo o conjunto de dados que dá suporte aos resultados deste estudo foi publicado no próprio artigo.

# Translation: Autocuidado individual y familiar de la salud genitourinaria de hombres hospitalizados: ¿intervienen los profesionales de enfermería?

**DOI:** 10.1590/1980-220X-REEUSP-2025-0135es

**Authors:** Carolina Andrade Costa, Osvaldo Conceição Portugal, Stefane Santos de Jesus Pitanga, Ariana Luiza Rabelo, Rafael Lima Rodrigues de Carvalho, Anderson Reis de Sousa

**Affiliations:** 1 Universidade Federal da Bahia, Escola de Enfermagem, Salvador, BA, Brasil.

**Keywords:** Autogestión, Sistema Urogenital, Enfermedades Urogenitales Masculinas, Salud del Hombre, Cuidados de Enfermería, Teorías de Enfermería

## Abstract

**Objetivo:**

Analizar el autocuidado individual/familiar de la salud genitourinaria de hombres hospitalizados y las intervenciones de enfermería evidenciadas.

**Método:**

Estudio cualitativo, de desarrollo tecnológico, mediante el Modelo Práctico para el Desarrollo de Tecnologías. Participaron 35 miembros: 19 pacientes varones, ocho familiares y ocho profesionales de enfermería. Se empleó la observación participante, el método de Análisis Temático de Contenido Reflexivo y el referencial de la Teoría del Autocuidado Individual y Familiar.

**Resultados:**

El autocuidado individual está marcado por la experiencia de la hospitalización, se ve debilitado en la vida cotidiana, restringe los cuidados de higiene íntima, busca asistencia médica centrada en la complejidad media/alta, fitoterapia automedicada, tolerancia a los síntomas, con empeoramiento de la condición clínica urológica. Existe apoyo familiar femenino, con resultados que promueven el bienestar e intervenciones de enfermería centradas en la realización de técnicas y procedimientos.

**Conclusión:**

El autocuidado de la salud genitourinaria por parte de los hombres se potencia con la experiencia de la hospitalización. Los hombres recurren y cuentan con el apoyo familiar durante la hospitalización, especialmente de sus parejas, cuyas intervenciones de enfermería no son específicas, individualizadas ni promotoras de la autogestión individual y familiar.

## INTRODUCCIÓN

La salud genitourinaria ha sido motivo de preocupación para la salud global, lo que representa un desafío cada vez mayor para las políticas públicas de salud y los sistemas de salud. La prevalencia de las enfermedades que afectan al tracto genitourinario ha aumentado significativamente, lo que repercute negativamente en importantes dimensiones de la calidad de vida y el bienestar, con consecuencias físicas, emocionales y sociales^(1,2)^.

Cabe recordar que el sistema genitourinario comprende una compleja red de componentes anatómicos y fisiológicos, integrando los sistemas urinario y reproductivo, entrelazándose con la salud sexual y la expresión de la sexualidad. Esencial para el mantenimiento de la vida, este sistema desempeña funciones primordiales como la gestión del metabolismo, la excreción de toxinas, la regulación del volumen corporal, la homeostasis hormonal y, además, las funciones sexuales y reproductivas.

Frecuentemente expuesto a diversas condiciones, el tracto genitourinario requiere atención y evaluación en los servicios de salud, particularmente en hombres cisgénero, ya que existe una amplia variedad de enfermedades y problemas ya documentados, como hiperplasia prostática, cáncer de próstata, prostatitis, infecciones genitales, enfermedad de Peyronie, cáncer de pene y testículos, insuficiencia renal, infecciones urinarias, litiasis, incontinencia urinaria, disfunciones sexuales, eyaculación precoz y otros problemas reproductivos asociados^(2,3,4,5)^.

La información epidemiológica sobre las afecciones genitourinarias es amplia, dado su contexto global, sin embargo, es importante acercarse a algunos datos. La literatura internacional señala que el 90% de los hombres entre 50 y 80 años viven con uno o más síntomas de problemas del tracto urinario inferior^(2)^. En Polonia, de 214 063 hospitalizaciones en el sector urológico, el 72% eran de sexo masculino^(5)^. En Asia, la incontinencia urinaria (IU) se señala como un factor de riesgo para la ansiedad, la depresión y las dificultades en el trabajo^(6)^. En Brasil, un estudio con 375 participantes varones reveló que el 51,1% tenía algún grado de disfunción eréctil y el 35,2% presentaba algún grado de síntomas del tracto urinario inferior, lo que se correlacionaba significativamente con la edad y la calidad de vida^(7)^.

A pesar de este panorama, una parte de la población masculina, a menudo influenciada por los patrones tradicionales de masculinidad, se muestra reacia a acceder a los servicios de salud, lo que implica la necesidad de estrategias para ampliar el acceso y el manejo de los problemas de salud de esta población^(6)^. En lo que respecta a la práctica de la enfermería, las implicaciones de la salud genitourinaria son profundas y multifacéticas, con amplias posibilidades de actuación, que pueden incluir desde la atención con enfermeros clínicos hasta la atención con enfermeros especializados. Cabe destacar que los enfermeros desempeñan un papel fundamental en la prevención, evaluación, gestión y rehabilitación de los trastornos genitourinarios, y que las acciones basadas en la evidencia tienen el potencial de mejorar significativamente los resultados de los pacientes, lo que puede potenciarse mediante el uso de innovaciones tecnológicas, con el fin de proporcionar una atención de calidad^(2)^.

Asumir el cuidado de uno mismo, junto con los familiares, es esencial para el desarrollo del autocuidado en favor de la calidad de la salud. Una de las formas en que se puede ayudar a la responsabilización del individuo como principal autor de su autocuidado es a través de una atención basada en la teoría del autocuidado. Esta teoría, desarrollada por las enfermeras Polly Ryan y Kathleen Sawin, afirma que las personas y las familias que se involucran en comportamientos de autocuidado mejoran los resultados de salud, ya que existe la necesidad de manejar las afecciones crónicas y participar activamente en un estilo de vida que promueva la salud^(8)^.

Existen lagunas en la literatura sobre cómo los hombres manejan la salud genitourinaria de manera no restrictiva a la enfermedad en el contexto hospitalario. Un estudio que aborde este tema puede ayudar a levantar indicadores de los comportamientos de salud masculina como fenómeno sensible a la práctica de la enfermería. Asimismo, es necesario ampliar la comprensión sobre los recursos individuales existentes y empleados por los hombres cuando se enfrentan a una hospitalización y a la experiencia de una enfermedad urológica, así como los de sus familiares y la inserción de los profesionales del campo de la enfermería, con el fin de comprender las intervenciones realizadas.

Ante lo expuesto, el objetivo de este artículo es analizar el autocuidado individual y familiar de la salud genitourinaria de los hombres hospitalizados desde la perspectiva de la participación de los profesionales del campo de la enfermería.

## MÉTODO

### Tipo de Estudio

Estudio cualitativo^(9,10)^, derivado de una tesis de maestría en Enfermería y Salud, con el fin de producir innovación tecnológica: elaboración de un nuevo producto, a través del Modelo Práctico para el Desarrollo de Tecnologías. Para ello, se trata del cumplimiento de la Fase Pragmática, configurada por: entrada en el campo práctico, observación/reflexión, comprensión/interpretación de la realidad vivida, conocimiento de los actores sociales, saberes y prácticas para la planificación de soluciones, mediante la secuencia de acciones: deducción; análisis; inducción; y síntesis derivada de los datos de la investigación de campo^(11)^. Este estudio se orientó por la pregunta de investigación: ¿Cómo se lleva a cabo el autocuidado individual y familiar de la salud genitourinaria por parte de los hombres hospitalizados? El diseño del estudio siguió las directrices del COREQ.

### Lugar

La investigación se llevó a cabo en una enfermería de urología de un hospital público universitario de la red federal en Bahía, Brasil. La unidad de referencia cuenta con 23 camas, distribuidas en 12 habitaciones, y atiende a pacientes de ambos sexos e identidades de género, siendo referencia para cirugías del proceso de transexualización, con demandas genitourinarias y ginecológicas. La sala presta servicios de enfermería, medicina, nutrición, fisioterapia, bioimagen y laboratorio. Las principales enfermedades y afecciones encontradas fueron: hiperplasia prostática, neoplasias malignas de vejiga, pene, próstata y litiasis renal.

### Población y Criterios de Selección

Los participantes en el estudio fueron hombres adultos/ancianos ingresados en el hospital con necesidades genitourinarias afectadas, familiares/acompañantes y profesionales del campo de la enfermería. Se asignaron los siguientes criterios de selección:

- Hombres mayores de 18 años, con necesidades de salud genitourinaria, atendidos en la unidad hospitalaria de urología. Criterios de exclusión: Hombres recién ingresados en la unidad de estudio, que presenten inestabilidad hemodinámica que imposibilite la realización de la entrevista;
- Familiares/acompañantes: ser miembro de la familia y/o cuidador durante la hospitalización. Criterios de exclusión: ser visitante;
- Profesionales de enfermería: trabajar en la enfermería de urología investigada. Criterios de exclusión: estar de vacaciones o ausente de sus funciones ocupacionales.

Hubo una negativa expresa de un paciente debido a manifestaciones dolorosas, de dos acompañantes, por no sentirse cómodos para realizar la entrevista, y de tres profesionales debido a la falta de tiempo.

### Recopilación de Datos

La recopilación de datos se inició en el periodo comprendido entre julio y septiembre de 2024, a través de un equipo de investigación compuesto por estudiantes de licenciatura, maestría y doctorado e investigadores doctores con experiencia en la temática/metodología, mediante capacitaciones en recopilación de datos, sin vínculo directo con los participantes. Mediante la observación participante^(12)^, guiada por un instrumento propio, que contenía los siguientes temas: 1. Características de funcionamiento del servicio; 2. Perfil y demandas de la clientela (actitudes, comportamientos y prácticas de autocuidado de la salud genitourinaria); 3. Actuación/práctica profesional del equipo de enfermería; 4. Interprofesionalidad, validado internamente en el grupo de investigación y sometido a prueba piloto el primer día de campo durante tres meses. Se buscó establecer relaciones con los actores sociales observados, obtener una comprensión profunda del fenómeno y las intervenciones de enfermería. Se completaron 21 guías de observación de campo.

En un segundo momento, se realizaron entrevistas individuales, en encuentros únicos, con una duración media de 50 minutos, con un total de 31 horas de material de audio con pacientes/familiares y profesionales del campo de la enfermería. Para ello, se utilizó un instrumento semiestructurado, validado internamente por los miembros del grupo de investigación y sometido a una prueba piloto con un grupo de cinco participantes. El instrumento se compuso de preguntas cerradas relacionadas con las características sociodemográficas, laborales y de situación de salud (hombres adultos hospitalizados), así como de preguntas abiertas relacionadas con el objeto investigado, a saber: Hombres: cuéntenos cómo gestiona el cuidado genital y urinario de su propia salud; Familiares: descríbame cómo participa en el cuidado de la salud del hombre al que acompaña; Profesionales: cuénteme cómo interviene en el cuidado de la salud genitourinaria de los hombres y la relación con sus familiares.

Las entrevistas se dieron por concluidas cuando se observó el cumplimiento de los criterios metodológicos del Análisis Temático Reflexivo, a saber: reconocimiento de los significados generados a partir de la interpretación de los datos recopilados^(13)^. El material recopilado y grabado se transcribió íntegramente,se codificó manualmente y se organizó, sistematizó e identificó en un único archivo con la ayuda de Google Drive^®^ y una hoja de cálculo en Excel^®^, para constituir el corpus de análisis. Se verificó la integridad, duplicidad e incompletitud de los datos.

### Análisis y Tratamiento de los Datos

Se adoptó el método de Análisis de Contenido Temático Reflexivo, ejecutado en seis fases: (1) Familiarización con los datos: inmersión mediante la lectura y relectura de los datos recopilados con el fin de identificar ideas iniciales y extraer etiquetas (157); (2) Generación de códigos iniciales: producción sistemática de códigos iniciales (58); (3) Búsqueda de temas: se agruparon los códigos en posibles temas (56); (4) Revisión de los temas: refinamiento de los temas identificados, verificando la representatividad y la coherencia interna; (5) Definición y denominación de los temas: se procedió al refinamiento de los temas, atribuyéndoles nombres y descripciones claras; (6) Elaboración del informe, redacción del artículo científico^(14,15)^. Se adoptaron medidas para lograr la fiabilidad en la investigación cualitativa: validación interna por parte del equipo de investigación y los miembros del grupo de investigación, evaluación externa por parte de expertos en la área y junto con los participantes^(16)^.

Se empleó la Teoría del Autocontrol Individual y Familiar para interpretar los resultados, la cual explica el autocontrol como un fenómeno multidimensional y complejo que afecta a individuos y familiares. El autocuidado se ha utilizado para referirse a tres fenómenos: el proceso, relacionado con las habilidades de autorregulación para manejar las condiciones crónicas o los factores de riesgo, como la definición de metas, el autocontrol y también el pensamiento reflexivo. El segundo fenómeno: programa/intervenciones, que se relaciona con la capacitación de las personas para asumir la responsabilidad de manejar sus propias enfermedades, así como participar en actividades de promoción, prevención y recuperación de la salud. El tercer y último fenómeno son los resultados obtenidos tras la participación del individuo en las acciones de autocontrol, relacionados con el proceso de mejora o estabilización de la condición de salud^(8)^.

### Aspectos Éticos

El proyecto fue aprobado bajo el dictamen CAAE: 76017423.3.3001.0049 y n.º: 6.821.769/2024, de conformidad con las Resoluciones 466/2012, 510/2016 y 580/2018 del Consejo Nacional de Salud. Se aplicó un Término de Consentimiento Libre e Informado para obtener el consentimiento de cada grupo de participantes. Para garantizar el anonimato de los participantes, se utilizó la identificación mediante siglas: H de hombre, F de familiar y PE de profesional del campo de la enfermería, seguida de la categoría ejercida y del número de orden de la entrevista: H01, F05 y PE - Enfermera.

## RESULTADOS

### Caracterización de los Participantes

El estudio estuvo compuesto por 19 hombres hospitalizados, ocho familiares/acompañantes y ocho profesionales del campo de la enfermería, lo que sumó un total de 35 participantes en el estudio.

Hombres adultos y ancianos hospitalizados (19): edad media de 59 años, en su mayoría de raza/color autodeclarado negro (11), estado civil casado (12), identidad de género cisgénero (19), orientación sexual heterosexual (19), nivel de estudios: bachillerato completo (6), con solo un participante con estudios superiores completos. En cuanto a la situación en el mercado laboral, la mayoría eran jubilados (14), y dos de ellos eran agricultores. En cuanto al salario medio, afirmaron «tener ingresos y contribuir al sustento de la familia» (14). Residen en su propia casa (18), todos ellos con acceso a electricidad y baño, sin embargo, 15 mencionaron tener agua corriente y 13 acceso a saneamiento básico, conviviendo en zona urbana (13) y zona rural (6), en presencia de sus parejas afectivas-conyugales (16). La mayoría tenía acceso a *Internet* (14) y teléfono celular (16).

Familiares/acompañantes (8): mujeres, siendo 6 esposas, 2 hijas, de raza/color negro (5), heterosexuales (8), cisgénero (8), sin ocupación (3), seguidas de jubiladas (2). Profesionales del campo de la enfermería (8): mujeres (7), raza/color negro (5), cisgénero (8), heterosexuales (8), formación superior (6) - Enfermera y formación técnica (2), con estudios de posgrado (5), sin embargo, ninguna en el área de Enfermería en Urología/Nefrología. Cinco de ellas tenían un único vínculo laboral, con una trayectoria profesional de más de 10 años de formación (7), sin experiencia profesional previa en el área de Urología, con una jornada laboral semanal de 36 horas.

### Hallazgos Empíricos del Fenómeno Investigado

Los resultados empíricos se presentaron en el marco teórico, considerando los siguientes aspectos: Elementos constitutivos de la Teoría del Autocuidado; Sustrato de la codificación de los datos empíricos y de la evidencia de los hallazgos empíricos, en lo que se refiere a: contexto del autocuidado; procesos de autogestión; resultados proximales y distales e intervenciones aparentes.

### Dimensión Individual del Autocuidado

#### Tema Generador Inicial: El Contexto del Autocuidado de la Salud Genitourinaria

El contexto del autocuidado de la salud genitourinaria realizado por hombres adultos hospitalizados se vincula con los cuidados básicos de higiene íntima, en la justificación de la ausencia de afecciones genitales, antecedentes familiares de enfermedades y afecciones genitourinarias, especialmente entre otros hombres de la familia, el mantenimiento del vínculo con un entorno considerado saludable, la evolución del empeoramiento de la condición clínica, lo que los lleva a intervenciones, procedimientos y hospitalización, y la percepción de apoyo por parte de los miembros de la familia.

Los familiares se vieron involucrados en un contexto en el que el autocuidado de la salud genitourinaria de los hombres hospitalizados exige tener que migrar de ciudad, recorrer itinerarios terapéuticos en el centro urbano, en un centro hospitalario de referencia, habiendo experimentado el acceso a otros servicios de salud disponibles en la red de atención en calidad de acompañantes, compuesta expresamente por mujeres, ya sean parejas sentimentales o conyugales, hijas y otras mujeres del ciclo familiar. Estas mujeres buscan cooperar con la promoción de un ambiente familiar saludable, incluyendo la enfermera del hospital, el apoyo a las medidas de tratamiento urológico empleadas por el equipo de salud, así como el cuidado de la alimentación y el uso de los medicamentos recetados. Además, expresaron su percepción sobre la persona significativa en relación con la salud y la experiencia de dificultades para desempeñar la función de acompañante. Así pues, la Cuadro 1 que figura a continuación presenta el marco teórico del contexto para la autogestión individual y familiar.

**Cuadro 1:** Marco teórico del contexto del autocuidado individual y familiar de la salud genitourinaria de hombres hospitalizados – Salvador, BA, Brasil, 2025.

Elementos constitutivos de la Teoría del Autocuidado	Sustrato de la codificación de los datos empíricos	Hallazgos empíricos
**Contexto:** Factores protectores: noción de higiene íntima; correlación del uso del jabón para la higiene íntima; entorno rural, doméstico y laboral; presencia y disponibilidad de los familiares. Riesgos: falta de avances en el cuidado de la higiene íntima debido a la ausencia de enfermedades o afecciones; presencia y recurrencia de infecciones urinarias; antecedentes de enfermedades genitourinarias en la familia (cáncer de próstata).		
**Condición: factores específicos; percepción individual/familiar** (Complejidad de la condición y el tratamiento, trayectoria, estabilidad de la condición y transiciones)	Tema: La higiene íntima con el uso de jabón común; Tema: Cuidados básicos de higiene íntima debido a la ausencia de problemas genitales; Tema: Lavado y uso de productos para la higiene del pene; Tema: Historial de eventos genitourinarios a lo largo de la vida; Tema: Progresividad del problema de salud genitourinaria; Tema: Sometimiento a procedimientos para resolver problemas urinarios; Tema: Multiplicidad de enfermedades y afecciones genitourinarias; Tema: Experiencia de eventos críticos de salud genitourinaria; Tema: Experiencia de complicaciones de la función urinaria/miccional; Tema: Trayectoria recorrida que implica la migración interestatal; Tema: Acompañamiento familiar en el traslado al hospital; Tema: Acompañamiento familiar en servicio ambulatorio especializado.	[...] *No utilizo ningún producto de higiene específico. Realizo cuidados comunes, como el uso de jabón, sobre todo porque tampoco tengo ningún problema genital. Nunca he tenido enfermedades antes.* (H01); [...] *mi problema de salud viene de muchos años atrás, me operaron hace muchos años y, hasta hoy, sigo teniendo muchas crisis. He tenido muchas infecciones urinarias y me sometí a una máquina para «romper los cálculos*» (se refiere a la realización del procedimiento de litotricia). (H05); [...] *Hay días en los que voy cuatro o cinco veces al baño a orinar. El problema es que la orina gotea cuando orino.* (H07); [...] *lo que me afectaba era tener que ir varias veces al baño a orinar.* (H08); [...] *nos derivaron desde el servicio municipal donde vivimos y llegamos hasta aquí* (se refiere al hospital de referencia del estado)*.*(F05); [...] él *tuvo acceso a la policlínica de mi región, donde vivo.* (F05).
**Entorno físico y social** (Acceso a la salud, transporte, cultura, capital social)	Tema: Ampliación del acceso al servicio de salud urológica; Tema: Afrontamiento de las dificultades en la asistencia hospitalaria en urología; Tema: Mantenimiento de la convivencia en el entorno rural; Tema: Mantenimiento de las actividades laborales; Tema: Promoción de un entorno saludable por parte de los familiares; Tema: Apoyo familiar en el tratamiento urológico.	[...] *el hospital necesita abrir sus puertas para que los hombres no tengamos dificultades, evitando que dejen de cuidarse por el hecho de tener dificultades para conseguir una consulta, programar el procedimiento, la plaza en el hospital y el transporte para desplazarse.* (H01); [...] *pienso seguir viviendo en el campo, realizando mi trabajo,* alimentándome bien*para vivir más tiempo.* (H03); [...] *cuido la alimentación, la casa, siempre trato de mantener el ambiente limpio, saludable, higiénico, la ropa limpia.* (F02); [...] *cuando surgió el problema de salud, empezamos a buscar tratamiento en la ciudad más cercana, pero no pudimos resolverlo, tuvimos que viajar y ser derivados al hospital de la capital.* (F05).
**Factores individuales y familiares** (Etapas de desarrollo, capacidad de aprendizaje, alfabetización, estructura y funcionamiento familiar, capacidad de autogestión)	Tema: Noción del compromiso de la salud genitourinaria por las infecciones de transmisión sexual; Tema: Percepción de que los hombres establecen múltiples relaciones sexuales; Tema: Conocimiento limitado de las enfermedades y afecciones genitourinarias; Tema: Práctica de una alimentación saludable como estrategia para el manejo de la función genitourinaria; Tema: Empleo de medidas de higiene genitourinaria y percepción del alcance de buenos resultados; Tema: Atribución de relaciones causales de complicaciones urinarias: cálculos renales; Tema: Antecedentes de enfermedades renales y genitourinarias entre los hombres de la familia; Tema: Mortalidad paterna y su relación con el cáncer de próstata; Tema: Apoyo familiar y protagonismo de las parejas afectivas y conyugales; Tema: Apoyo familiar en la prestación de cuidados alimenticios y médicos; Tema: Percepción familiar del estado de salud de la persona significativa; Tema: Dificultades para adquirir los conocimientos necesarios para cuidar al familiar.	[...] *Sé que algunas enfermedades transmisibles pueden causar problemas en los* órganos *genitales y urinarios, como el cáncer, en el caso de los hombres que tienen muchas parejas sexuales.* (H01); [...] *He oído hablar de las infecciones urinarias* [...] *Mi padre tuvo una infección urinaria.* (H03); [...] *trato de no comer alimentos grasos, de no engordar. Además, tengo cuidado con la sonda vesical, evito tocarla en el suelo, la dejo colgando para evitar que las bacterias lleguen a la vejiga.* (H04); [...] *me lavo bien el pene y lo limpio después de orinar* [...] *la gente dice que los cálculos renales se deben a la falta de líquido y yo bebo mucha agua* [...] *tengo dos hermanos que utilizan un drenaje en el riñón.*(H05); [...] *necesito cuidarme, procurar estar limpio* [...] *mi papá tuvo antes de morir una enfermedad de la próstata, la próstata se alteró hasta el punto de no poder orinar.* (H06); [...] *tengo el apoyo de mi familia. Mi esposa se ofreció y tuvo la buena voluntad de quedarse conmigo y cuidarme. Tenemos una buena convivencia, a pesar del poco tiempo que llevamos juntos.* (H07); [...] *creo que hago correctamente mi higiene* íntima*.* (H08); [...] *bebo poca agua, pero voy a beber más. Utilizo jabón para la higiene* íntima [...] *dos hermanos tuvieron problemas de próstata*. (H09); [...] *tengo una familia numerosa y, por eso, siempre tengo a alguien con quien contar para que me ayude a cuidar de mi salud, sobre todo ahora que estoy hospitalizado y he tenido que someterme a una cirugía.* (H10); [...] *yo le doy los medicamentos, yo le preparo la comida, pero* él *puede bañarse y hacer otras cosas por sí mismo.* (F03); [...] *no tenía conocimiento de su problema de salud.* Solo *me enteré después de la hospitalización.* (F06); [...] *le han diagnosticado una enfermedad renal y de próstata, pero en la próstata no necesita intervención por ahora. He oído hablar de la enfermedad renal, pero confieso que no tengo los conocimientos suficientes para cuidarlo.* (F07).

El proceso de autogestión de la salud genitourinaria empleado por hombres adultos hospitalizados expresó (Figura 1),conocimientos y creencias relacionados con las causas de las enfermedades genitourinarias, con énfasis en las infecciones de transmisión sexual, el uso de fitoterápicos, a menudo recomendados por personas cercanas, de uso propio y sin indicación de un profesional de la salud.

**Figure 1:** FModelo explicativo de la aplicación de la Teoría del Autocuidado Individual y Familiar de la salud genitourinaria de hombres adultos hospitalizados. Salvador, BA, Brasil.

Fuente: Adaptado de Ryan, Sawin, 2009.

Surgieron metas establecidas para el momento actual de la hospitalización y el futuro, con una perspectiva de mejora de la actividad física, la alimentación, el uso de medicamentos y el autocuidado. Además, se manifestaron mecanismos de autocontrol de los síntomas y molestias genitourinarias, como la autoinspección y la vigilancia de la eliminación urinaria. También se pusieron de manifiesto pensamientos reflexivos sobre su estado de salud actual y las posibilidades de replantearse los hábitos y prácticas de cuidado de la salud genitourinaria, graduando y/o nivelando su estado de salud. Para apoyar el autocuidado, los familiares hicieron referencia a Dios, expresando percepciones sobre la relación entre el familiar y el cuidado de su salud, impregnada por el contexto de agravamiento de la condición de salud genitourinaria y la aparición de complicaciones, vinculadas a las estrategias de apoyo que se brindan a los hombres. Además, los familiares tuvieron que lidiar con la aprensión de la cirugía, la búsqueda de la facilitación del autocuidado, en la medida en que expresaron afecto por el familiar enfermo, demostrando dedicación y atribuyendo valor al cuidado del otro, en su mayoría, parejas afectivas-conyugales. Así pues, la Cuadro 2 que figura a continuación presenta el marco teórico del proceso de autogestión para la autogestión individual y familiar.

**Cuadro 2:** Marco teórico del proceso de autogestión del autocuidado individual y familiar de la salud genitourinaria de los hombres hospitalizados – Salvador, BA, Brasil, 2025.

Elementos constitutivos de la Teoría del Autocuidado	Sustrato de la codificación de los datos empíricos	Hallazgos empíricos
**Proceso de autocuidado**		
**Conocimientos y creencias** (Información objetiva, autoeficacia, expectativa de resultados, congruencia de objetivos)	Tema: Atribución de las relaciones entre la masculinidad y la salud genitourinaria; Tema: Adhesión a los medicamentos fitoterapéuticos por recomendación de personas cercanas; Tema: Adopción de comportamientos evasivos para evitar complicaciones de salud genitourinaria; Tema: Concepción de la salud más allá de la enfermedad y búsqueda ampliada de profesionales médicos de diversas especialidades; Tema: Atribución de la franja de edad como marco para establecer medidas de autocuidado de la salud y la enfermedad; Tema: Búsqueda de un médico urólogo; Tema: Referencia a Dios como beneficioso y promotor del consuelo, demostrando servirle.	[...] *Hay muchos hombres que no usan condón durante las relaciones sexuales, hacen todo lo que quieren. Por eso surgen problemas como el HTLV, el VIH, la sífilis y todo eso compromete la función genital y urinaria.* (H02); [...] *Tomar té para eliminar cálculos, mandacaru u otros que me recomiendan.* (H05); [...] estoy tratando de cuidar mi salud para que los problemas no se acumulen, si no será mucho peor, no puedo parar. (H07); [...] la salud es prevención, necesito cuidarme aunque no esté enfermo, ir al médico, no solo al urólogo, sino también a otros especialistas. (H08); [...] *tomo los cuidados normales. Si es necesario utilizar el medicamento, lo utilizo;* (H10); [..] *a partir de los 40, 45 años, el hombre tiene que acudir al urólogo* [...] *desde que enfermé, busqué ser siervo de Dios, tuve problemas en el pasado que me perjudicaban y no me dejaban bien. Fue entonces cuando escuché la palabra de Dios, que me sirvió de consuelo.* (H11). [...] *gracias a Dios me sentí bien al verlo regresar de la cirugía* [...] *seguiré pidiendo a Dios que le conceda salud.* (F05).
**Competencias y habilidades de autorregulación** (Establecimiento de metas, autocontrol y reflexión, toma de decisiones, planificación y acción, autoevaluación, control emocional)	Tema: Búsqueda de la longevidad a partir de una alimentación saludable, actividad física y ocio; Tema: Reflexiones sobre uno mismo y su estado de salud actual y proyecciones futuras; Tema: Cumplimiento de la terapia médica relacionada con la medicalización; Tema: Atribución de parámetros de autoevaluación de la salud; Tema: Adopción de comportamientos de alerta para el consumo de agua; Tema: Identificación de síntomas urinarios desagradables; Tema: Inspección de los órganos genitales con mención al pene y los testículos; Tema: Explicación de las características de comportamiento y rasgos de personalidad relacionados con el cuidado de la salud; Tema: Manifestación de tolerancia al dolor y otros síntomas; Tema: Revelar la percepción de la relación del familiar con la salud; Tema: Revelar la gravedad del problema de salud genitourinaria del familiar y las estrategias de apoyo proporcionadas.	[...] *intento hacer ejercicio físico, leer, ver qué es lo mejor.* (H01); [...] *si no me cuido yo, ¿quién lo hará?*. (H06); [...] *me alimento bien, tomo la medicación, hago lo que me dicen los médicos* [...] *si no me cuido ahora, ¿qué pasará mañana? Hoy tengo un 90% del flujo urinario. Si dejo de cuidarme mañana, ¿cómo voy a estar?* (H04); [...] *me estoy cuidando, hoy en* día *tenemos que cuidarnos, lo mandó el médico.* [...] *estoy atento para beber mucha agua. Lo que más suelo hacer ahora es seguir la recomendación de beber mucho líquido. Noto mal olor en la orina cuando no bebo mucha agua.* (H05); [...] *miro cómo están mis testículos.* (H07); [...] *los parientes de mi familia suelen llegar a los 100 años y yo también pretendo llegar a esa edad sin necesitar la ayuda de nadie.* (H10); [...] *a veces soy terco, tardo en ir al servicio de salud* [...] *soy un poco testarudo, sentía dolor y me quedaba mucho tiempo en casa resistiéndome, no era muy disciplinado, pero ahora necesito mantener la disciplina* [...]*con todo lo que me ha pasado, me está abriendo los ojos, mi cuerpo me dio una señal, el cuerpo nos da señales, ¿no? Me quedaba mucho tiempo en casa preguntándome: ¿se me pasará este dolor? Ahora estoy reflexionando sobre mí mismo, sobre mi salud, digamos así... la buena salud es cuando estás al 100%. La mía, veo que está al 40%.* (H11); [...] *le cuesta ir al médico, no le gusta. Cuando la familia descubrió que tenía este problema, enseguida empezamos a buscar medios, a buscar un médico.* (HA05).
**Facilitación social** (Influencia social, apoyo emocional, instrumental o informativo, colaboración negociada)	Tema: Acompañamiento familiar durante la hospitalización urológica; Tema: Protagonismo de las esposas como acompañantes en la unidad hospitalaria; Tema: Asimilación de la información sanitaria proporcionada por los profesionales médicos; Tema: Entre la aprensión por la cirugía y el alivio de tener al familiar cerca; Tema: Dedicación familiar al cuidado; Tema: Valoración del cuidado prestado al familiar esposo.	[...] *mi familia siempre me ha acompañado. Mi esposa me ha acompañado hasta aquí, al hospital.* (H07); [...] *sobre enfermedades como el cáncer y la infección urinaria, siempre veo a los médicos hablando.* (H11); [...] *me sentí nerviosa en el momento de la cirugía, pero todo salió bien y hoy* él *está bien, hospitalizado, recibiendo mi apoyo.* (HA05); [...] *le dedico todo mi tiempo, le cuido todo el* día, *todos los* días, con la comida, la *ropa y otras cosas, le recuerdo que tome la medicación y le acompaño al médico.* (HA06); [...] *es mi esposo, tengo que cuidarle y acompañarle lo mejor posible, y de la mejor manera que puedo.* (HA07).

Los resultados que surgieron del autocuidado de la salud genitourinaria empleado por hombres adultos hospitalizados revelaron, en una dimensión próxima, la búsqueda de asistencia médica, con la presencia de la pareja afectiva-conyugal, la aparición de la figura de la enfermera como promotora de la educación en salud, el acceso a servicios de salud de urgencia y emergencia, y la adhesión a prácticas integradoras y complementarias relacionadas con los fitoterápicos. Ante el contexto de los resultados distales obtenidos en la experiencia del autocuidado de la salud genitourinaria, los familiares expresaron afinidad por el cuidado y la sensación de bienestar al realizar esta tarea, que se entrelaza con la manifestación de cansancio, sobrecarga y preocupaciones por el familiar hospitalizado (Cuadro 3), lo que suscita intervenciones de enfermería, que se concentraron en el manejo clínico y la ejecución de procedimientos de enfermería, con una intervención educativa y promotora del autocuidado individual y familiar limitada (Cuadro 4).

**Cuadro 3:** Marco teórico de los resultados proximales y distales del autocuidado individual y familiar de la salud genitourinaria de los hombres hospitalizados – Salvador, BA, Brasil, 2025.

Elementos constitutivos de la Teoría del Autocuidado	Sustrato de la codificación de los datos empíricos	Hallazgos empíricos
**Resultados proximales y distales**		
**Resultados proximales: comportamientos de autogestión individual y familiar** (Participación en actividades/regímenes de tratamiento, uso de terapias farmacológicas, gestión de síntomas)	Tema: Seguimiento médico como actividad/régimen de tratamiento genitourinario; Tema: Presencia femenina como promotora de la salud de los hombres con enfermedades genitourinarias; Tema: Enfermera como profesional que enseña, orienta y da capacitación; Tema: Automedicación y uso de fitoterápicos como prácticas; Tema: Búsqueda de un profesional médico en situación secundaria a los intentos de automanejo en el domicilio; Tema: Búsqueda de servicios de urgencia y emergencia; Tema: El profesional médico como punto focal de primera búsqueda; Tema: Uso de medicamentos como recurso terapéutico para el manejo de los síntomas.	[...] *en mi caso fue el seguimiento médico de mi esposa y mío. Busco asistencia médica.* (H01); [...] *siempre he acudido a los médicos para que me orienten. Siempre es bueno recibir orientación de los médicos* [...] *la enfermera me enseña y me orienta para que haga los ejercicios aquí en el hospital.* (H02); [...] tomaba medicamentos por mi cuenta, usaba una planta y preparaba mi té. No tomaba medicamentos. Tomaba una hoja de boldo, la cocinaba y la tomaba para aliviarme, y cuando la situación se agravaba, buscaba un médico. (H03); [...] busco cuidarme porque el médico me lo ordenó. (H04); [...] *consumo líquido, té de hoja de quebrapedra y caña de macaco do brejo* [...] *cuando no se resuelve, acudo al médico.* (H05); [...] buscaba un médico, y eso ocurría en la UPA*. Tomo medicamentos como dipirona y té de boldo, hierba santa, hierba limón, cuando estoy en casa. (H06); [...] *fui directamente al médico en la clínica.* (H07); [...] *ya he tenido que usar medicamentos por mi cuenta.* (H10). ^*^Unidad de Atención de Urgencias 24 horas.
**Resultados proximales: costo de los servicios de salud** (El costo asociado al uso de la atención médica)	Tema: Adopción de medidas consideradas saludables ante la imposibilidad de sufragar otros recursos. Tema: Necesidad de asistencia farmacéutica. Tema: Desembolso de recursos financieros con la aseguradora.	[...] *hoy busco la mejor manera posible de llevar una vida saludable, ya que no puedo pagar un seguro médico.* (H09);[...] *ya he necesitado tomar medicamentos y ahora pago un seguro de salud*. (H10).
**Resultados distales: estado de salud** (Prevención, mitigación, estabilización)	Tema: Establecimiento de cuidados genitales preventivos; Tema: Adopción de cuidados de higiene con dispositivo urinario; Tema: Uso de fitoterápicos en forma de tés como solución para aliviar los síntomas; Tema: Búsqueda de asistencia médica con periodicidad; Tema: Práctica de la automedicación como recurso terapéutico.	[...] *gozo de buena salud, ya que mantengo una buena higiene, el pene siempre está limpio y la sonda vesical también.*(H04); [...] *el problema de mi riñón se solucionó tomando té. Cuando tomaba té, se me solucionaba y me sentía bien.*(H06); [...] *ahora voy al médico, pero antes, cuando me dolía algo, tomaba un medicamento.* (H09).
**Resultados distales: calidad de vida** (Bienestar percibido, costo de la salud)	Tema: Satisfacción en la búsqueda de mejorar la salud y obtener ayuda de los demás; Tema: Percepción de bienestar al cuidar de la propia salud; Tema: Sentimiento de gratitud vinculado al vínculo familiar; Tema: Sentimiento de optimismo y utilidad; Tema: Sentimiento de realización vinculado a la cohesión familiar; Tema: Percepción de la priorización de la propia salud; Tema: Afinidad por el cuidado y sentimiento de bienestar en el manejo de la saludenfermedad del familiar.	[...] *Estoy satisfecho buscando mejorar. Voy madurando y buscando lo mejor para mí.* (H01); [...] *Me siento satisfecho de tener gente que me ayuda.* (H03); [...] *Me siento bien, me hace bien y necesito cuidarme por mi bienestar.* (H05); [...] *los tés que tomaba me hacían sentir bien. Me siento bien cuidando mi salud. Estoy agradecido por la familia que tengo y que me hace sentir bien.* (H06); [...] *es genial cuidarme a mí mismo y saber que soy* útil*.* (H09); [...] *me siento realizado con el apoyo familiar que tengo para cuidar mi salud.* (H10); [...] *me siento bien porque estoy cuidando mi salud, que ahora es una prioridad.* (H11); [...] *cuando cuido de* él *es porque me gusta verlo bien y eso me hace sentir bien a mí también.* (HA02); [...] *me siento bien porque me llevo bien con* él*, gracias a Dios, es un esposo estupendo, así que tengo que cuidarlo.* (A05).
**Resultados distales: costo de la salud** (Costo directo e indirecto)	Tema: Cancelación de medidas para mantener la atención médica; Tema: Desembolso de recursos financieros para mantener la salud; Tema: Cansancio, sobrecarga y preocupaciones experimentadas por los familiares.	[...] *tenía seguro, pero como era caro lo cancelé*. (H09); [...] *pago un seguro para que venga a visitarme*. (H10); [...] *es agotador, porque mi papá necesita cuidados todo el tiempo*. *Estábamos preocupadas por el diagnóstico y su estado de salud. Y, en particular, yo estaba ansiosa por acompañarlo.* (HA03); [...] *tengo que hacerlo por* él*, a veces me siento muy cansada, sobrecargada. La cirugía tardó mucho y me preocupé.* (A04); [...] *tenía miedo, y cuanto más tiempo pasaba sin que regresara del quirófano, más desesperada me sentía. Regresó por la noche* (A05).

**Cuadro 4:** Marco teórico de las intervenciones centradas en el autocuidado individual y familiar de la salud genitourinaria de los hombres hospitalizados – Salvador, BA, Brasil, 2025.

Elementos constitutivos de la Teoría del Autocuidado	Sustrato de la codificación de los datos empíricos	Hallazgos empíricos
**Intervenciones**		
**Intervenciones centradas en el individuo y la familia**	Tema: Ejecución de técnicas y procedimientos de enfermería; Tema: Control de la eliminación urinaria mediante irrigación vesical; Tema: Desobstrucción de la sonda; Tema: Examen físico del aparato genital; Tema: Control del balance hídrico; Tema: Control de episodios hemorrágicos; Tema: Establecimiento de un diálogo para ampliar los conocimientos sobre salud de los pacientes varones.	[...] *en la medicación, el cambio de aparatos y dispositivos.* (P01); [...] *realizar curas de heridas infectadas, utilizando productos como carbón activado, alginato* [...] *irrigación vesical, control de hemorragias, registros de enfermería.* (P02); [...] *administración de medicamentos, cuidados con los drenajes, eliminación de secreciones, irrigación vesical.* (P03); [...] *controlar de manera eficaz el equilibrio hídrico, las irrigaciones vesicales.* (P04); [...] *Intento hablar con los pacientes para que conozcan su estado de salud.* (P05); [...] *Realizo la desobstrucción de la sonda vesical, principalmente cuando el paciente regresa del quirófano* [...] *examen físico de los genitales.* (P05).

#### Dimensión Profesional del Autocuidado: Profesionales del Campo de la Enfermería

## DISCUSIÓN

Los resultados de este estudio revelaron un predominio de individuos negros y morenos, cisgénero, heterosexuales, casados, con diferentes niveles de escolaridad y en su mayoría jubilados, con una media de 59 años. Es fundamental tener en cuenta el perfil de los hombres entrevistados para analizar el autocuidado individual y familiar de la salud genitourinaria de estos, ya que dichas características pueden influir en el proceso de cuidado.

En lo que respecta al contexto del autocuidado de la salud genitourinaria, entre los factores protectores y de riesgo, se destacaron los cuidados básicos de higiene íntima, los antecedentes familiares de enfermedades y afecciones genitourinarias, la figura femenina como promotora del cuidado, con enfoque en las esposas, el mantenimiento de un entorno considerado saludable, y la falta de avances en la higiene íntima vinculada a la ausencia de enfermedades como factores de riesgo. Los factores contextuales explicados en la teoría contribuyen a una comprensión más amplia de cómo se lleva a cabo el autocuidado en el ámbito individual y en la díada intrafamiliar o en la unidad familiar. Estos factores contextuales pueden afectar al autocuidado y comprometer el proceso, dadas las dimensiones contextuales y procesales existentes en la gestión de la salud, especialmente cuando se vive con la funcionalidad genitourinaria afectada. Cuando las intervenciones de enfermería tienen en cuenta el contexto, pueden contribuir a la reducción de los riesgos para la salud y promover condiciones favorables para el autocuidado, lo que se puede evidenciar ampliamente en nuestros hallazgos^(8)^.

En lo que respecta a la higiene íntima ampliamente mencionada, los hallazgos en la literatura indican que los conocimientos masculinos asociados al cuidado del cuerpo suelen estar relacionados con la noción de higiene corporal, con especial importancia a los órganos genitales, asociada a la inspección continua de estos^(17)^. Sin embargo, es importante destacar que el cuidado genitourinario no debe limitarse únicamente a los órganos genitales, sino que es necesario un cuidado más amplio e integrado en la salud general.

En este estudio fue evidente la ausencia de hombres como cuidadores, mientras que la presencia femenina surge como promotora de la salud de los hombres y protagonista del autocuidado familiar, lo que puede correlacionarse con cuestiones socioculturales, en las que el cuidado presenta un escaso reconocimiento social y una fuerte feminización^(18)^. Las mujeres, a menudo esposas e hijas, son corresponsables de la gestión de los cuidados, especialmente en presencia de un ser querido enfermo, y se ven invadidas por sentimientos que implican el bienestar por cuidar y manejar la salud-enfermedad del familiar, pero también por la responsabilidad moral, la obligación, además de las dificultades con la sobrecarga, el cansancio y las preocupaciones por el familiar hospitalizado^(18)^.

En el proceso de autogestión de la salud genitourinaria se evidenció la influencia de los conocimientos y las creencias, con el uso constante de fitoterápicos, la automedicación y el establecimiento de metas con una perspectiva de mejora. Los resultados del presente estudio presentan los componentes notorios del proceso de autogestión, lo que puede ayudar a comprenderlos resultados, ya sean proximales o distales del manejo de la salud genitourinaria^(8)^. El uso de fitoterápicos, a menudo recomendados por vecinos, amigos y familiares, presenta matices complejos. El uso de plantas medicinales para promover la salud y/o complementar los tratamientos de determinadas afecciones debe considerarse con cautela, ya que su uso no está exento de riesgos^(19)^. Estos hallazgos pueden explicarse mediante la Teoría del Autocuidado, según la cual las intervenciones de enfermería dirigidas al proceso de autocuidado contribuyen a mejorar los conocimientos y las creencias en materia de salud, además de ayudar a aumentar la adopción de comportamientos de autorregulación de sí mismo y de la salud por parte del individuo, facilitando la socialización^(8)^.

Además, a pesar de que Brasil cuenta con una regulación a través de la Política Nacional de Plantas Medicinales y Fitoterapéuticos y la incluye en las Prácticas Integrativas y Complementarias del SUS, los estudios indican un conocimiento erróneo en varios aspectos importantes, como los efectos adversos de esta terapia, el desconocimiento de las sustancias químicamente activas y el desconocimiento de las interacciones medicamentosas^(19,20,21)^. En este contexto, es fundamental explorar conocimientos multidisciplinarios e interdisciplinarios que involucren el conocimiento popular y científico, en la búsqueda de estrategias que promuevan el bienestar y reduzcan o eliminen la posibilidad de daños al usuario y el agravamiento de una determinada afección^(19)^.

En los resultados proximales y distales, se utilizó la figura médica como referencia, la búsqueda secundaria del profesional médico, el desembolso de recursos financieros y la dificultad de acceso al servicio especializado. Cabe señalar que, en la Teoría del Autocuidado Individual y Familiar, la influencia social se concibe como un mensaje o diálogo en el que personas en posición de autoridad, respetadas y con conocimientos especializados, aconsejan y fomentan comportamientos saludables^(8)^. Así, el médico mencionado repetidamente encaja en este papel, ya que es visto como el profesional de primera elección y como impulsor de la atención, en la que algunos pacientes realizan determinadas prácticas de salud únicamente porque han sido orientados por el médico, lo que puede ser un reflejo de una atención médica centrada en el médico. Además, refleja la importancia de la relación médico-paciente, dado su papel fundamental en la prevención, el diagnóstico y el tratamiento de la enfermedad, así como en el éxito terapéutico^(19,22)^.

En lo que respecta a la búsqueda de servicios de salud, existe una relación íntima con los patrones tradicionales de masculinidad. Las normas socioculturales desempeñan un papel significativo en la renuencia de los hombres a buscar servicios de salud, a menudo por temor a parecer débiles, por el estigma y la discriminación, además de la escasez de profesionales masculinos^(23,24)^. Cabe destacar también la presencia del edadismo en los discursos, en los que se cita una delimitación de la franja de edad como marco para establecer medidas de autocuidado de la salud y la enfermedad. Cabe señalar que este fenómeno puede estar influenciado por la edad, el género, el nivel educativo y el miedo a la muerte^(25)^.

El acceso a los servicios de salud es determinante para el alcance terapéutico. En este estudio se evidenciaron obstáculos que impactan en este acceso, tales como itinerarios terapéuticos lejanos recorridos por los hombres y sus familiares que residen en municipios distantes de la capital, barreras en el acceso a los servicios ante la complejidad de programar consultas y procedimientos, junto con la disponibilidad de plazas en el hospital. Corroborando estos hallazgos, un estudio que analizó las barreras de acceso en cinco regiones de Brasil evidenció dificultades relacionadas con la accesibilidad geográfica, asociadas a la falta de disponibilidad de consultas especializadas en la propia Región de Salud, la insuficiencia de plazas de hospitalización, los largos tiempos de espera y la integración deficiente o inexistente de los servicios^(26)^. A esto se suma el hecho de que determinados problemas genitourinarios, como la incontinencia, pueden generar impactos multidimensionales, afectando no solo a la calidad de vida de los pacientes, sino también a la estructura, el proceso y los resultados de la atención^(27)^.

Los resultados revelan que la dimensión profesional del autocuidado en lo que respecta a la actuación de los profesionales del campo de la enfermería se centró en la ejecución de técnicas y procedimientos asistenciales, sin que se produjeran intervenciones de enfermería centradas en la educación o la aplicación de recursos tecnológicos, como en el caso de la indicación de aplicaciones disponibles para el manejo de problemas de salud genitourinarios derivados del cáncer de próstata y de las cirugías de prostatectomía radical^(28)^. La teoría del autocuidado puede ser útil para orientar las intervenciones de enfermería individuales y centradas en la familia, ya que tienen un impacto positivo en el autocuidado de la salud, especialmente cuando tienen en cuenta el contexto y el proceso^(8)^. Lo mismo ocurre con los recursos de apoyo diagnóstico/terapéutico de la incontinencia por parte de los enfermeros^(29)^. Por lo tanto, el establecimiento de medidas, programas centrados en la autonomía, habilidades, establecimiento de metas, transición de la atención, rehabilitación, preparación y planificación para el alta hospitalaria, así como la escasez de intervenciones específicas para la promoción y el mantenimiento de la salud genitourinaria en el contexto hospitalario investigado y con implicaciones en el autocuidado^(30)^.

Cabe destacar también que las profesionales de enfermería investigadas no tenían formación ni especialización en el área de Enfermería en Urología, lo que puede implicar una actuación poco amplia y un debilitamiento de la asistencia de enfermería en la atención ampliada de las necesidades de salud genitourinaria. Es urgente y necesario realizar esfuerzos en el ámbito de la formación en Enfermería en Salud Masculina, Enfermería Nefrológica y Urología, así como fortalecer los procesos de trabajo en enfermería, en la aplicación del Proceso de Enfermería y las Teorías de Enfermería, que apoyen la toma de decisiones clínicas de los equipos de enfermería que trabajan en este segmento. Sin embargo, se recomienda cautela en la comprensión de los conceptos esenciales y los constructos de las dimensiones del autocuidado de la salud genitourinaria por parte de los hombres, considerando que los resultados alcanzados por los hombres pueden verse fácilmente afectados por las dimensiones del contexto y del proceso. Esto exigirá a los profesionales de enfermería una mayor inversión en la evaluación clínica de enfermería (anamnesis, examen clínico): reducción de la morbimortalidad, control de las tasas metabólicas, prevención y manejo de afecciones crónicas, uso preciso de medicamentos, con miras a mejorar el estado de salud a partir de la autogestión^(8)^.

Asimismo, de los profesionales del campo de la enfermería que trabajan en otros puntos de la red de atención primaria de salud, como lo corrobora un estudio anterior que evidencia las contribuciones de la actuación del enfermero en el tratamiento de la disfunción del tracto urinario inferior^(29)^. En este sentido, se recomienda prestar atención a lo que señala la teoría del autocontrol. Esta teoría indica que las construcciones basadas en el contexto, el proceso y los resultados combinados entre sí de forma única pueden implicar avances sustanciales para la práctica clínica del autocuidado de la enfermedad crónica por parte de los hombres afectados, haciéndolos más comprometidos con la adopción de comportamientos específicos en relación con la salud y la enfermedad genitourinaria^(8)^.

Este estudio presenta potencialidades e implicaciones para la producción de conocimiento y su traducción y para la práctica profesional en enfermería: analiza y explica el fenómeno del autocuidado; presenta aspectos particulares y singulares de género/masculinidades en la relación entre el cuidado y la autogestión de la salud por parte de los hombres; fortalece los estudios clínico-sociales en el contexto de la atención hospitalaria; explora la necesidad genitourinaria de salud como foco y agenda de investigación; enumera los elementos constitutivos del autocuidado, que resultan útiles para orientar/guiar el razonamiento clínico/diagnóstico del profesional de enfermería y orientar al equipo de enfermería en la producción de cuidados genitourinarios de salud. Además, se ha demostrado el potencial práctico de una Teoría de Enfermería de Alcance Medio, que ha resultado adecuada para la especificidad investigada y que puede explorarse en futuras investigaciones e intervenciones asistenciales y educativas^(8)^.

Este estudio presenta las siguientes limitaciones: la técnica y el método empleados involucraron a grupos distintos, lo que puede requerir el uso de recursos diversificados y específicos para una comprensión más profunda, explicativa y reflexiva del fenómeno investigado, lo que puede limitar la capacidad de abstracción, así como la comprensión de los patrones, propiedades y dimensiones de los datos encontrados. El hecho de que la investigación se haya realizado en el contexto clínico-asistencial hospitalario puede haber reducido la capacidad de abstracción por parte de los profesionales del campo de la enfermería, ante las dispersiones, las múltiples atenciones y demandas del proceso de trabajo, así como la censura de determinada información sensible y relevante que se debía recabar de los participantes varones y sus familiares/acompañantes, lo cual debe tenerse en cuenta.

## CONCLUSIÓN

Los elementos constitutivos del autocuidado individual y familiar se encontraron en la experiencia de hombres con necesidades de salud genitourinarias afectadas durante la hospitalización y favorecen el avance en la práctica clínica de la enfermería al revelar el contexto y el proceso en el que y cómo se produce el autocuidado, los resultados que se obtienen en la interacción con la familia y el alcance, la amplitud y las orientaciones de las intervenciones de enfermería que se implementan, lo que pone de manifiesto indicios de ampliación, fortalecimiento y especificidad. En el ámbito de la dimensión individual, el contexto está marcado por la experiencia de la hospitalización, con un autocuidado de la salud genitourinaria debilitado en la vida cotidiana. Las prácticas de cuidado se vinculan a la higiene íntima, la asistencia médica centrada en el médico y el aumento de alternativas de fitoterapia automedicada. Búsqueda de servicios de salud de media y alta complejidad ante el empeoramiento de la condición clínica urológica, teniendo la presencia femenina como punto de referencia familiar de apoyo.

El proceso de autogestión se basa en creencias relacionadas con la salud y la enfermedad genitourinaria, el establecimiento de metas para restablecer la condición de salud y el funcionamiento genitourinario, impregnadas de autorreflexiones y autoevaluación de la salud, que se suman a las experiencias psicoemocionales de los familiares que los acompañan durante la hospitalización. Los resultados proximales y distales indican la adopción de comportamientos de autogestión, sin embargo, movilizados por el tratamiento y las terapias centradas en el médico, que repercuten en los costos para ellos y para el sistema de salud, pero que expresan una mejora en la calidad de vida por parte de los hombres encuestados.

La actuación familiar es significativa en el contexto del autocuidado en lo que respecta a los factores específicos y la percepción sobre el familiar hospitalizado, cuyo funcionamiento familiar expresa cohesión y apoyo, pero está impregnado de dificultades en cuanto al conocimiento sobre el proceso de salud-enfermedad genitourinaria. Existe la creencia familiar de que Dios concede consuelo, cuyas competencias y habilidades de autorregulación revelaron la resistencia de los hombres en el manejo autónomo de su salud y el agravamiento de los problemas de salud genitourinaria, lo que exige el empleo de estrategias de apoyo a los hombres con funcionalidad genitourinaria afectada. El resultado del autocuidado apoyado por los familiares implica beneficios para ellos mismos, pero no apunta a contribuciones proximales para los hombres.

## DISPONIBILIDAD DE DATOS

Todo el conjunto de datos que apoya los resultados de este estudio fue publicado en el propio artículo.
